# Comparative efficacy and safety of dual-combination vs. triple-combination antihypertensive therapies in hypertensive patients: an updated meta-analysis of randomized controlled trials

**DOI:** 10.3389/fphar.2026.1786728

**Published:** 2026-05-15

**Authors:** Zhuoling Gong, Lijun Huang, Xinru Long, Yan Zhang

**Affiliations:** 1 Jiangxi University of Traditional Chinese Medicine, Nanchang, China; 2 The Affiliated Hospital of Jiangxi University of Traditional Chinese Medicine, Nanchang, China

**Keywords:** hypertension, antihypertensive drugs, dual-combination, triple-combination, meta-analysis

## Abstract

**Background:**

Combination therapy is often required for treating hypertension,but the comparative efficacy and safety of triple versus dual antihypertensive regimens remain to be clarified by the latest evidence-based medical data. This meta-analysis aims to compare the efficacy and safety of dual versus triple antihypertensive drug therapy for adult primary hypertension, providing updated evidence to support clinical decision-making.

**Methods:**

A systematic search was conducted across four databases—PubMed, Embase, Cochrane, and Web of Science—up to July 2025 to identify randomized controlled trials comparing dual antihypertensive therapy with triple antihypertensive therapy for treating adult primary hypertension. The primary outcome was mean seated blood pressure; secondary outcomes included blood pressure control rates and adverse events. Screening, data extraction, and quality assessment were performed by two independent researchers.The Cochrane Risk of Bias Assessment Tool was used to evaluate study quality; data analysis was conducted using Stata 15.1 software.

**Results:**

A total of 21 studies involving 17,669 patients were ultimately included. Of these, 6,918 patients received triple therapy and 10,751 patients received dual therapy. Triple therapy reduced systolic blood pressure [WMD: −5.98 mmHg, (95% CI: −7.04, −4.92)] and diastolic blood pressure [WMD: −3.34 mmHg, (95% CI: −4.03, −2.65)]. In the ARB subgroup, valsartan-based triple therapy demonstrated significant blood pressure-lowering effects on systolic blood pressure [WMD = −7.41 mmHg, (95% CI: −8.91 to −5.91)] and diastolic blood pressure [WMD = −4.57 mmHg, (95% CI: −5.65 to −3.49)]. An evaluation of 14 adverse reaction symptoms revealed that triple therapy improved patient fatigue [RR: 0.80, (95% CI: 0.67, 0.96)]. Triple therapy increased the rate of blood pressure control [RR: 1.31, (95% CI: 1.23, 1.39)].

**Conclusion:**

Compared with the dual-drug regimen, the triple-drug antihypertensive regimen may be more effective for short-term blood pressure control, and its overall safety profile is acceptable. However, the subgroup analyses in this study represent exploratory findings only and cannot be directly used to guide clinical drug selection. Given that, although the evidence for the primary efficacy outcome is of moderate certainty, significant heterogeneity remains, the results should be interpreted with caution.

## Background

Hypertension is regarded as one of the most substantial global health challenges, with a strong association to the onset of cardiovascular, metabolic, and cognitive diseases (Wei et al., 2025). According to data released by the World Health Organization (WHO) in 2023,54% of adults with hypertension worldwide have been diagnosed, 42% are receiving treatment, and only 21% have their hypertension under control (Kario et al., 2024).

A study conducted in the United States has indicated that in excess of 20% of hypertensive patients also have concomitant diabetes and hyperlipidaemia. Moreover, it has been determined that merely 25% of adults afflicted with hypertension, diabetes, and hyperlipidaemia have their conditions adequately manage (Lee et al., 2025). The risk factors for hypertension are diverse, including genetics, age, metabolism, unhealthy lifestyle habits, living environment, and comorbid conditions (Yusuf et al., 2020). The interplay of these factors collectively exacerbates the management of blood pressure, thereby imposing a considerable health and economic burden on society and families. In low- and middle-income countries, a paucity of healthcare resources and inadequate health management awareness means that only 8% of patients achieve blood pressure control, which subjects them to considerable financial strain (Schutte et al., 2021). At present, treatment strategies for hypertension primarily consist of a comprehensive approach combining non-pharmacological interventions with drug therapy. Non-pharmacological interventions primarily encompass nursing care, behavioural and psychological interventions, as well as physical and lifestyle interventions (Zou et al., 2025). With regard to the utilisation of pharmaceutical interventions, it is a widely acknowledged fact that monotherapy is only efficacious for a minority of patients, frequently fails to achieve sustained blood pressure control, and may result in an increased incidence of adverse reactions when administered at high doses (Rosas-Peralta et al., 2025). Consequently, the majority of patients require two to three medications to effectively manage blood pressure. The European Society of Cardiology (ESC) (McEvoy et al., 2024) and the American College of Cardiology/American Heart Association (ACC/AHA) (Greenland and Peterson, 2017) both emphasise the significance of combination antihypertensive therapy. In the extant literature, dual therapy regimens combining two drugs with different mechanisms of action have been demonstrated to be efficacious in reducing blood pressure (Prabhakaran et al., 2025). Nevertheless, a considerable proportion of patients continue to exhibit suboptimal blood pressure levels following the administration of dual therapy (Higaki et al., 2017). For this group of patients, the pivotal clinical decision concerning blood pressure management is whether to adjust the dosage and switch medication classes to optimise the regimen or to directly apply triple therapy.Research studies (Lacourcière et al., 2012; Oparil et al., 2010) have indicated that in cases where blood pressure remains uncontrolled following a period of 4 weeks of adequate or appropriate dual therapy, the subsequent escalation to triple therapy may be a viable consideration. In comparison with dual therapy, triple therapy has been shown to achieve earlier and more potent blood pressure reduction through multi-target synergistic effects, improving control rates and potentially further lowering overall cardiovascular risk. It is evident that common triple-combination regimens generally comprise a calcium channel blocker (CCB), a renin-angiotensin system inhibitor (e.g., an angiotensin receptor blocker [ARB] or an angiotensin-converting enzyme inhibitor [ACEI]), and a diuretic. ARBs, such as valsartan, losartan, olmesartan, telmisartan, azilsartan, and the renin inhibitor aliskiren, are frequently employed in combination with amlodipine (CCB) and hydrochlorothiazide or chlorthalidone (diuretics) in triple therapy regimens.

In recent years, a substantial body of high-quality RCT data has been accumulating, evaluating the comparative efficacy of dual versus triple antihypertensive regimens. It is possible that previously published meta-analyses may not have incorporated this new evidence. The objective of this study is to synthesise the most recent evidence from randomised controlled trials that is available through July 2025 via meta-analysis methods. The objective of this study is to compare the efficacy and safety of dual versus triple antihypertensive regimens in adults with primary hypertension. The findings of this study will provide more targeted and individualised treatment decision-making guidance for clinical practice.

## Methods

### Literature search

A predefined retrieval framework was established using the keywords “Hypertension,” “Blood Pressure, High,” “High Blood Pressure,” “Blood Pressures, High,” “High Blood Pressures,” “Triple Therapy,” “Triple Combination,” “Three-drug Combination,” “Three Drug Therapy,” “Triple Drug Therapy,” “Quadruple,” “Multi drug,” “fixed dose,” “Fixed combination,” “Drug Therapy, Combination,” “Dual Therapy,” “Dual Combination,” “Two-drug Combination,” “Two Drug Therapy,” “Dual Drug Therapy,” “Dual,” and other keywords. A systematic search of the literature was conducted using the PubMed, Embase, Cochrane, and Web of Science databases, with the search period extending up to 15 July2025.

### Inclusion and exclusion criteria

Studies must meet the following inclusion criteria: (Wei et al., 2025): Study type: randomized controlled trial; (Kario et al., 2024); Participants diagnosed with primary hypertension aged≥18 years (Lee et al., 2025); Treatment intervention: triple-drug versus dual-drug therapy (Yusuf et al., 2020); Primary outcome: mean sitting blood pressure; secondary outcomes: blood pressure control rate and adverse events.

Exclusion criteria (Wei et al., 2025): Studies were reviews, subgroup analyses, etc*.*, (Kario et al., 2024); Key indicators or safety outcome measures were missing; (Lee et al., 2025); Only pooled data for patients with primary hypertension were reported, rendering the data unusable (Yusuf et al., 2020); Duplicate publications or conference abstracts with incomplete information; (Schutte et al., 2021); Study subjects had comorbid conditions.Studies meeting any of these criteria were excluded.

### Data extraction

Data extraction was performed independently by two researchers using a standardized template. Any discrepancies were resolved through discussion, with a third researcher intervening when necessary. The detailed information collected included: first author, year of publication, country, sample size, gender ratio, mean age, intervention group, intervention outcome, baseline blood pressure, and duration of treatment.

### Quality assessment

The Cochrane Risk of Bias Tool was utilised to evaluate the risk of bias and methodological quality of the included randomised controlled trials. In accordance with the recommendations set forth by the Cochrane Collaboration, the evaluation of studies was conducted across seven domains: random sequence generation, allocation concealment, blinding of outcome assessors, blinding of study participants, incomplete data reporting, selective reporting, and other biases. Each study was subjected to a rigorous examination process prior to the allocation of a risk of bias rating of “low risk,” “unclear risk,” or “high risk.” The assessments were conducted by two independent researchers, with any disagreements resolved through discussion. In instances of discordance, a third researcher was engaged to achieve consensus.

### Data analysis

Statistical analysis was performed using Stata version 15.1. A 95% confidence interval (CI) was calculated. For dichotomous data, the risk ratio (RR) was used to measure effect size, while for continuous variables, the weighted mean difference (WMD) was used. The I^2^ statistic was used to assess heterogeneity; if I^2^>50% or p < 0.1, indicating significant heterogeneity, a random-effects model was used; otherwise, a fixed-effects model was employed. Funnel plots and Egger’s regression analysis were used to assess potential publication bias. The Trim and Fill method was used to evaluate the impact of publication bias on the interpretation of results. If I^2^ > 50%, sensitivity analyses were conducted to assess the robustness of the results, and meta-regression analyses were performed on data with high heterogeneity regarding triple drug combinations, dual drug combinations, treatment duration, baseline systolic blood pressure,and baseline diastolic blood pressure. P < 0.05 was considered statistically significant. Additionally, subgroup analyses were conducted based on different types of ARB drugs.

### Evidence quality grading

The GRADE approach was used to conduct an overall assessment of the evidence for each outcome. GRADE assigns a certainty rating of high, moderate, low, or very low based on the degree of confidence that the estimated effect is close to the true effect. It uses five criteria: risk of bias in individual studies, inconsistency, indirectness, imprecision, and publication bias. Data from RevMan version 5.4 were imported into the GRADE analyzer (GRADEpro) to derive the results.

## Results

### Literature search flowchart

**FIGURE 1:**
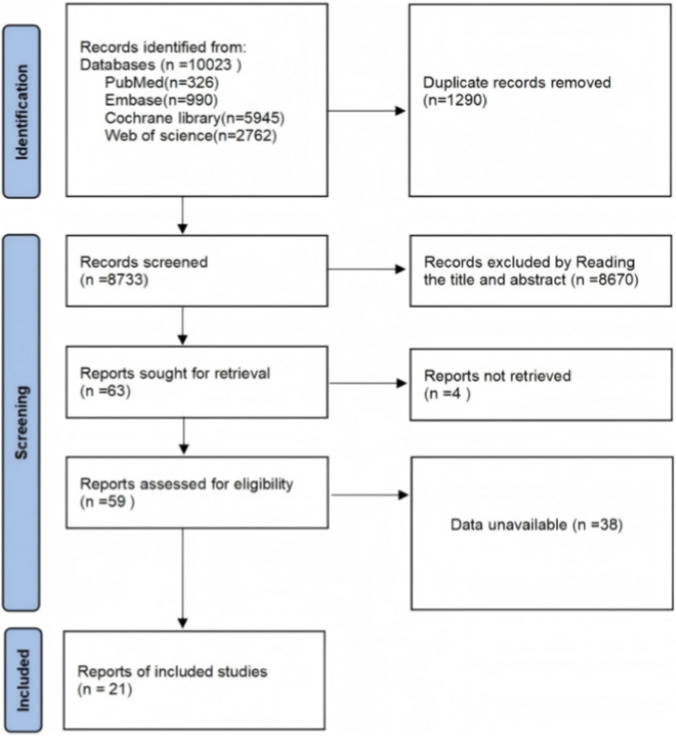
Literature search flowchart.

A total of 10,023 articles were retrieved through database searches: 326 from PubMed, 990 from Embase, 5,945 from the Cochrane Library, and 2,762 from Web of Science. After removing 1,290 duplicates, 8,670 articles were excluded based on title and abstract screening. Four original articles were unavailable, and 38 lacked data. Ultimately, 21 articles were included. Title and abstract screening was conducted independently by two researchers, who recorded eligible records. Full texts of potentially eligible records were retrieved and assessed independently by both researchers. Discrepancies were resolved through discussion, with a third researcher providing final adjudication when necessary. Detailed screening procedures and specific exclusion criteria for each stage are outlined in the PRISMA 2020 flowchart (Figure 1).

### Basic characteristics table

Baseline study results included demographic and clinical characteristics of the enrolled population (see Table 1). This analysis encompassed 21 studies (Higaki et al., 2017; Lacourcière et al., 2012; Oparil et al., 2010; Rodgers et al., 2024; Bhushan et al., 2014; Sugimoto et al., 2013; Calhoun et al., 2009; Cho et al., 2023; Cranfield et al., 1988; Mapesi et al., 2025; Rakugi et al., 2018; Hong et al., 2017; Ferdinand et al., 2011; Sung et al., 2023; Rump et al., 2016; Volpe et al., 2012; Destro et al., 2010; Rakugi et al., 2014; Rakugi et al., 2015; Sung et al., 2018; Sohn et al., 2016) involving 17,669 patients, all of whom were individuals with poorly controlled primary hypertension or stage 2 hypertension and above. Among these, 6,918 patients received triple therapy and 10,751 patients received dual therapy. The included studies were published between 1988 and 2025. The final selection comprised six studies from the Americas, five from Europe, and 10 from Asia. The overall proportion of male patients was higher. The mean age was 56.6 years in the triple therapy group and 56.9 years in the dual therapy group. The triple-drug combination regimen primarily comprised calcium channel blockers (CCBs) + angiotensin II receptor blockers (ARBs) +diuretics, compared against corresponding dual-drug combinations. ARBs included losartan, valsartan, olmesartan, telmisartan, azilsartan; CCBs were predominantly amlodipine; Hydrochlorothiazide was the predominant diuretic, though some studies used chlorthalidone (Hong et al., 2017; Sung et al., 2023) or indapamide (Rodgers et al., 2024). Only one study of dual therapy did not include drugs present in the triple combination (Cranfield et al., 1988).

**TABLE 1:** Basic characteristics table.

Study	Year	Country	Sample size		Gender (M/F)	Mean age		Intervention		Outcomes	Baseline blood pressure	Duration of intervention
			EG	CG		EG	CG	EG	CG			
Rodgers	2024	Australia	551	834	673/712	59	59	Telmisartan20 mg + Rmlodipine2.5 mg + Indapamide1.25 mg	① Telmisartan20 mg + Indapamide1.25 mg ② Telmisartan20 mg + Amlodipine2.5 mg ③ Amlodipine2.5 mg + Indapamide1.25 mg	F3; F17	The systolic blood pressure (SBP) ranging from 140 to 179 mmHg (on no drugs) to 110–150 mmHg (on three drugs)	12 weeks
Bhushan	2014	India	28	60	55/33	50	51	Ramipril2.5 mg + Telmisartan20 mg + Hydrochlorothiazide12.5 mg	① Ramipril5mg + Hydrochlorothiazide12.5 mg ② Telmisartan40 mg + Hydrochlorothiazide12.5 mg	F3; F4; F5	Stage 2 hypertensive patients	24 weeks
Sugimoto	2013	USA	574	1728	1218/1084	55.1	55.1	Olmesartan medoxomil40 mg +Amlodipine besylate10 mg +Hydrochlorothiazide25 mg	① Olmesartan medoxomil40 mg +Hydrochlorothiazide25 mg ② Olmesartan medoxomil40 mg +Amlodipine besylate10 mg ③ Amlodipine besylate10 mg +Hydrochlorothiazide25 mg	F3; F6; F7; F8; F9; F10; F11	Mean SeBP140/100 mmHg or 160/90 mmHg (off Antihypertensive medication)	12 weeks
Calhoun	2009	USA	583	1688	1255/1016	53.3	53.1	Valsartan320 mg + Hydrochlorothiazide25 mg + Amlodipine10 mg	① Valsartan320 mg + Amlodipine10 mg ② Hydrochlorothiazide25 mg + Amlodipine10 mg ③ Valsartan320 mg + Hydrochlorothiazide25 mg	F1; F2; F3; F7; F9; F10; F11; F12; F13; F14; F17	Mean SeSBP≥145 mmHg; mean SeDBP≥100 mmHg	12 weeks
Eun Joo Cho	2023	Korea	186	188	293/81	61.5	60.3	Telmisartan80 mg + Amlodipine5mg + Chlorthalidone25 mg	Telmisartan80 mg + Amlodipine5 mg	F1; F2; F7	The patients of 140 mmHg ≤ mean sitting Systolic BP < 200 mmHg	8 weeks
CRANFIELD	1988	England	102	99	74/127	58	56	Moducren® (Hydrochlorothiazide25 mg + Amiloride hydrochloride2.5 mg + Timololmaleate10 mg)	Spiroprop® (Spironolactone50 mg + Propranolol hydrochloride 80 mg)	F3; F7; F9; F11; F12; F17	The systolic and diastolic blood pressures were≥ 145 mmHg and/or 95 mmHg	12 weeks
Mapesi	2025	Switzerland	253	510	228/535	≥18 years		Amlodipine2.5 mg + Hydrochlorothiazide6.25 mg + losartan12.5 mg	Amlodipine5mg + Losartan50 mg	F1; F2; F17	Uncomplicated, untreated hypertension (standardized office blood pressure 140 mmHg systolic or 90 mmHg diastolic)	12 weeks
Rakugi	2018	Japan	142	67	139/70	56.7	57.0	① Azilsartan20 mg + Amlodipine5mg + Hydrochlorothiazide12.5 mg ② Azilsartan20 mg + Amlodipine5mg + Hydrochlorothiazide6.25 mg	Azilsartan20 mg + Amlodipine 5 mg	F1; F2; F3; F7; F8; F14; F16; F17	Office blood pressure (BP)≥ 150/95 mmHg	10 weeks
Sohn	2016	Korea	167	171	235/103	56.1	56.4	Olmesartan medoxomil20 mg +Amlodipine5mg + Hydrochlorothiazide12.5 mg	Olmesartan medoxomil20 mg +Hydrochlorothiazide12.5 mg	F1; F2; F4; F5; F7; F9; F10; F15; F17	Stage 2 hypertensive patients	8 weeks
Higaki	2017	Japan	149	160	257/52	54.4	55.0	Telmisartan80 mg + Amlodipine5mg + Hydrochlorothiazide12.5 mg	Telmisartan80 mg + Amlodipine5 mg	F1; F2; F16; F17	Had essential hypertension, were already taking two or three antihypertensive drugs , had a mean seated DBP ⩾90 and ⩽114 mmHg	8 weeks
Ferdinand	2011	USA	202	209	204/207	≥18 years		Aliskiren300 mg + Amlodipine10 mg + Hydrochlorothiazide25 mg	Aliskiren300 mg + Amlodipine10 mg	F1; F2; F3; F4; F7; F10; F13; F14; F17	US minority patients with stage 2 hypertension	8 weeks
Sung	2023	Korea	62	181	153/90	61.8	61.8	Amlodipine1.67 mg + Losartan potassium16.67 mg + Chlorthalidone 4.17 mg	① Amlodipine1.67 mg + losartan potassium16.67 mg ② Losartan potassium16.67 mg +Chlorthalidone4.17 mg ③ Amlodipine1.67 mg + Chlorthalidone4.17 mg	F1; F2; F3; F7; F10; F11	Mean SeSBP <180 mmHg and mean SeDBP <110 mmHg for participants who were already taking antihypertensive drugs or the mean SeSBP ≥140 to <180 mmHg and mean SeDBP <110 mmHg for participants who were not taking antihypertensive drugs	8 weeks
Rump	2016	Germany	539	269	469/339	55.7	55.9	① Olmesartan medoxomil40 mg +Amlodipine10 mg + Hydrochlorothiazide12.5 mg ② Olmesartan medoxomil40 mg +Amlodipine10 mg + Hydrochlorothiazide25 mg	Olmesartan Medoxomil40 mg +Amlodipine10 mg	F1; F2; F3; F5; F8; F10; F17	Has not taken the medication Mean SeSBP ≥160 mmHg and a mean SeDBP ≥100 mmHg Criteria for patients receiving a stable dose of antihypertensive monotherapy for 4 weeks prior to screening were a mean SeSBP≥150 mmHg and mean SeDBP≥95 mmHg	16 weeks
Volpe	2012	Italy	1680	1010	1247/1443	≥18 years		① Olmesartan20 mg + Amlodipine5mg VS Olmesartan20 mg + Amlodipine5mg + Hydrochlorothiazide12.5 mg ② Olmesartan40 mg + Amlodipine5mg VS Olmesartan40 mg + Amlodipine5mg + Hydrochlorothiazide12.5 mg and Olmesartan40 mg + Amlodipine5mg + Hydrochlorothiazide25 mg ③ Oimesartan40 mg + Amlodipine10 mg VS Olmesartan40 mg + Amlodipine10 mg + Hydrochlorothiazide12.5 mg and 0lmesartan40 mg + Amlodipine10 mg + Hydrochlorothiazide25 mg		F1; F2; F3; F5; F7; F10; F17	Mean SeBP (SeSBP/SeDBP)≥160/100 mmHg; a difference in mean seated BP (SeBP) of <20/10 mmHg between visits	10 weeks
Destro	2010	USA	136	208	179/165	≥18 years		Amlodipine10 mg + Valsartan160 mg + Hydrochlorothiazide 12.5 mg	Amlodipine10 mg + Hydrochlorothiazide 12.5 mg	F1; F2; F3; F4; F6; F7; F8; F10; F12; F14	Stage 2 hypertensive patients	8 weeks
Oparil	2010	USA	627	1865	1318/1174	54.7	55.2	Olmesartan Medoxomil40 mg +Amlodipine Besylate10 mg +Hydrochlorothiazide25 mg	① Olmesartan Medoxomil40 mg +Hydrochlorothiazide25 mg ② Olmesartan Medoxomil40 mg +Amlodipine Besylate10 mg ③ Amlodipine Besylate10 mg +Hydrochlorothiazide25 mg	F1; F2; F3; F4; F5; F6; F7; F8; F9; F10; F11; F13; F14; F17	Mean seated BP (SeBP) ≥140/100 or ≥160/90 mmHg (off treatment)	12 weeks
Lacourcie` re	2012	Canada	310	881	720/471	55.4	55	Aliskiren300 mg + amlodipine10 mg + hydrochlorothiazide25 mg	① Aliskiren300 mg + Hydrochlorothiazide25 mg ② Aliskiren300 mg + Amlodipine10 mg ③ Amlodipine10 mg + Hydrochlorothiazide25 mg	F1; F2; F3; F4; F7; F9; F10; F14; F17	msSBP≥160 and <200 mmHg and/or msDBP ≥100 and <120 mmHg t	8 weeks
Hong	2017	Korea	167	161	261/67	59.3	61.2	Amlodipine5mg + losartan potassium100 mg + chlorthalidone25 mg	Amlodipine5mg + losartan potassium100 mg	F1; F2; F3; F5; F7; F9; F15; F16	Stage 2 hypertensive patients	8 weeks
Rakugi	2015	Japan	164	163	253/74	54.9	55.4	Losartan 50 mg + Hydrochlorothiazide 12.5 mg +Amlodipine 5 mg	Losartan 50 mg + Amlodipine 5 mg	F1; F2; F5; F7; F10	DBP≥90 and <110 mmHg; SBP≥140 and <200 mmHg	8 weeks
Sung	2018	Korea	155	155	245/65	62	63.4	Telmisartan80 mg + Amlodipine10 mg + Hydrochlorothiazide25 mg	Telmisartan80 mg + Amlodipine10 mg	F1; F2; F3; F4; F7; F10; F11; F14; F15	Mean sitting systolic [MSS] BP of ≥140 and <200 mmHg	8 weeks
Rakugi	2014	Japan	141	144	218/67	53.9	56.3	Losartan 50 mg + amlodipine 5 mg + hydrochlorothiazide 12.5 mg	losartan50 mg +hydrochlorothiazide12.5 mg	F1; F2; F5; F7; F10	Sitting diastolic blood pressure (DBP) ≥90 mmHg but <110 mmHg, and a mean trough sitting systolic blood Pressure (SBP) ≥140 mmHg but <200 mmHg	8 weeks

EG: experimental group.

CG: control group.

F1: SeSBP; F2: SeDBP; F3: event-Headache; F4: event-cough; F5: event-Hypotension; F6:event-Hypokalemia; F7: event-dizziness; F8:event-Upper respiratory; F9: event-Fatigue; F10: event-Peripheral edema; F11: event-Nausea; F12: event-Dyspepsia; F13: event-Muscle spasms; F14: event-Nasopharyngitis; F15:event-Constipation; F16: event-Blood uric acid increased; F17: BP, control rate (SeSBP/SeDBP≤140/90 mmHg).

### Quality assessment diagram

**FIGURE 2:**
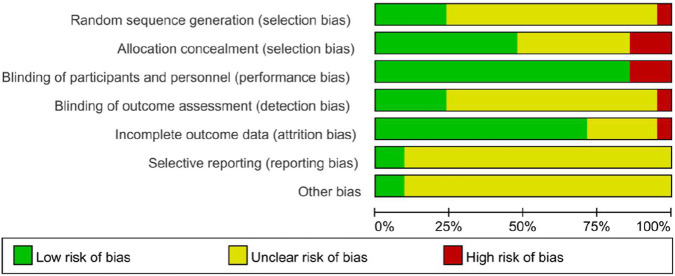
Risk of bias graph.

**FIGURE 3:**
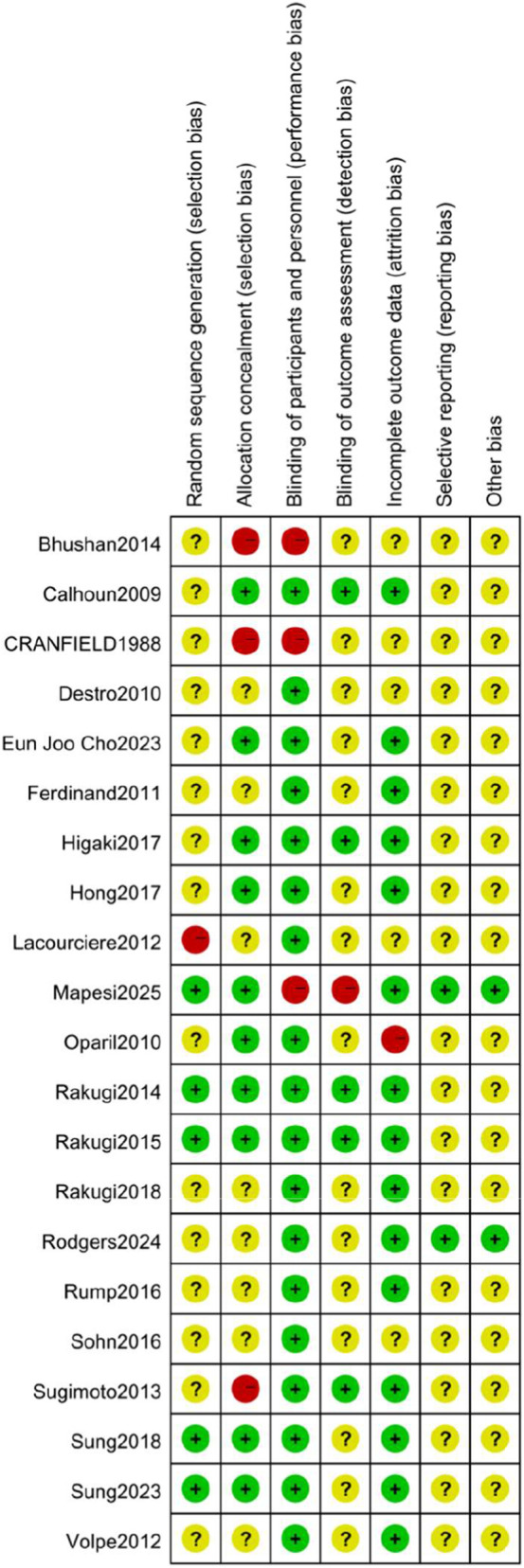
Risk bias of summary.

The risk of bias and methodological quality of included randomized controlled trials were assessed using the Cochrane risk of bias tool. Data were entered into RevMan 5.4 software to generate risk of bias plots and summary risk of bias plots (Figures 2, 3). Among the 21 studies, five explicitly described random sequence generation methods, employing either permutated block allocation (Mapesi et al., 2025; Rakugi et al., 2014; Rakugi et al., 2015; Sung et al., 2018) or computer programs (Sung et al., 2023). Three studies did not use double-blinding: two were open-label (Bhushan et al., 2014; Mapesi et al., 2025) and one employed single-blinding (Cranfield et al., 1988). Only two studies provided protocols confirming the absence of other biases (Rodgers et al., 2024; Mapesi et al., 2025). A GRADE assessment was conducted for systolic blood pressure, diastolic blood pressure, blood pressure control rates, and adverse events (Table 2). The results showed that the certainty of evidence for systolic blood pressure, diastolic blood pressure, and blood pressure control rates was moderate, primarily due to significant heterogeneity among studies and the risk of bias in some studies; the overall certainty of evidence for adverse event outcomes was low, mainly due to the small number of included studies, imprecise effect estimates, and design flaws in some studies.

**TABLE 2:** GRADE.

Outcome	Effect size (95% CI)	Participants (studies)	Certainty of evidence (GRADE)	Interpretation
Systolic BP (SBP)	WMD -5.98 (−7.04, −4.92)	17,669 (21 RCTs)	Moderate (●●●○)	Triple therapy reduces SBP
Diastolic BP (DBP)	WMD -3.34 (−4.03, −2.65)	17,669 (21 RCTs)	Moderate (●●●○)	Triple therapy reduces DBP
BP control rate	RR 1.31 (1.23, 1.39)	17,669 (21 RCTs)	Moderate (●●●○)	Improved control rate
Adverse events	Mixed results	17,669 (21 RCTs)	Low (●●○○)	Some risks increased

### SBP

**FIGURE 4:**
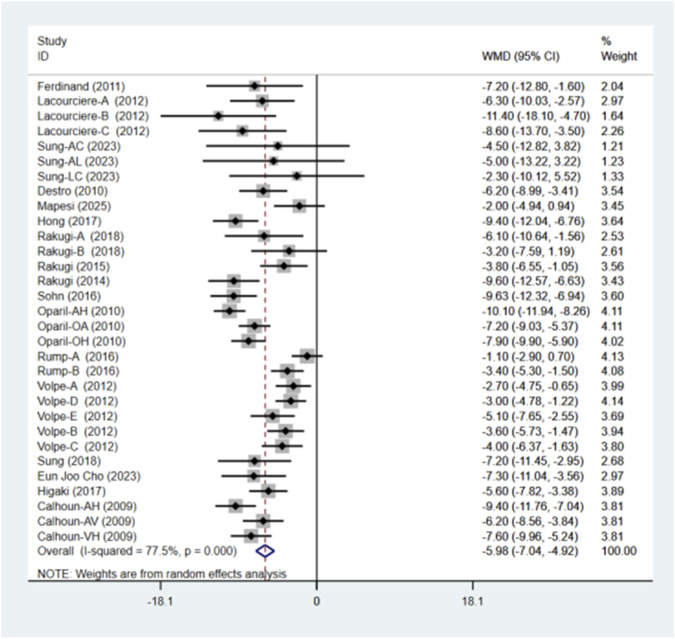
SBP forest plot.

**FIGURE 5:**
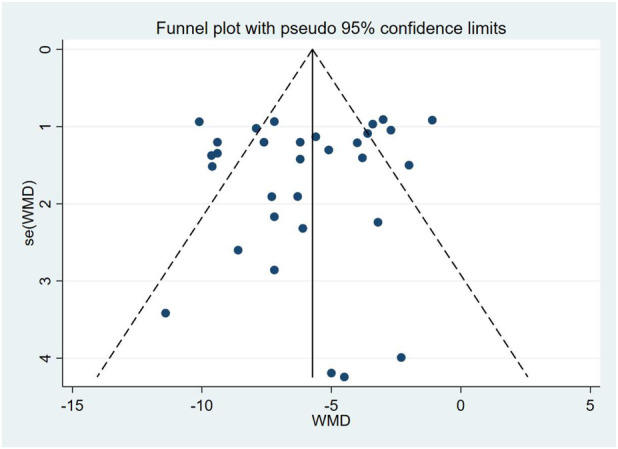
SBP funnel graph.

**FIGURE 6:**
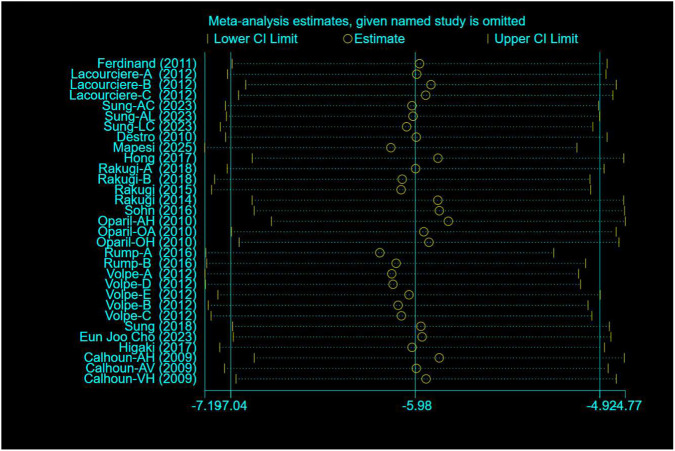
SBP sensitivity analysis.

**FIGURE 7:**
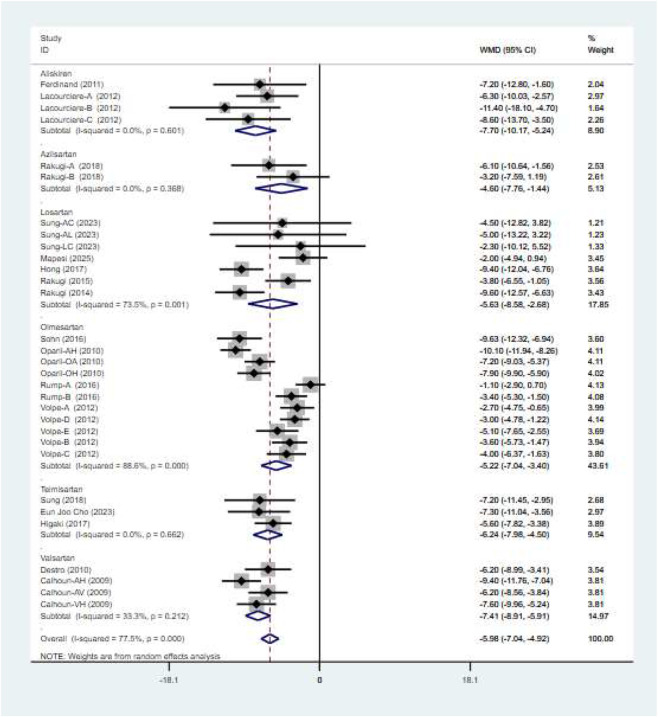
SBP-subgroups.

A total of 17 studies comprising 31 randomized controlled trials were included, comparing the blood pressure-lowering effects of triple versus dual antihypertensive therapy on SBP. Due to high study heterogeneity (I^2^ = 77.5%, P = 0.000), a random-effects model was employed for analysis. Results (Figure 4) showed that triple therapy significantly outperformed dual therapy in reducing SBP [WMD = −5.98, (95% CI: −7.04, −4.92)]. Publication bias assessment revealed a non-significant Egger test (P = 0.431), and the funnel plot (Figure 5) exhibited overall symmetry, indicating no substantial publication bias. Due to considerable heterogeneity in SBP reduction, sensitivity analysis was conducted by sequentially excluding studies. Results (Figure 6) demonstrated robust findings unaffected by individual studies. To further explore the potential sources of heterogeneity and assess the impact of different study characteristics on the blood pressure-lowering efficacy, a meta-regression analysis was performed. The results showed that the triple drug combinations (Table 3), baseline systolic blood pressure (Table 4), and diastolic blood pressure (Table 5) all had no statistically significant effect on heterogeneity; however, among the dual drug combinations (Table 6), “Amlodipine + Losartan” (p = 0.014), “Amlodipine + Olmesartan” (P = 0.010), and “Amlodipine + Olmesartan Medoxomil” (P = 0.002) may be the causes of increased heterogeneity; treatment duration (Table 7) of 8 weeks (P = 0.001) and 12 weeks (P = 0.001) may also be factors contributing to high heterogeneity. Most triple-drug regimens consist of a calcium channel blocker (CCB) + an angiotensin receptor blocker (ARB) + a diuretic; therefore, we classified subgroups based on ARB type into five categories: valsartan (Calhoun et al., 2009; Destro et al., 2010), losartan (Mapesi et al., 2025; Hong et al., 2017; Sung et al., 2023; Rakugi et al., 2014; Rakugi et al., 2015), olmesartan (Oparil et al., 2010; Sugimoto et al., 2013; Rump et al., 2016; Volpe et al., 2012; Sohn et al., 2016), telmisartan (Higaki et al., 2017; Rodgers et al., 2024; Bhushan et al., 2014; Cho et al., 2023; Sung et al., 2018), and azilsartan (Rakugi et al., 2018). Across all subgroups (Figure 7), triple-drug regimens containing valsartan [WMD = −7.41 mmHg, (95% CI: −8.91 to −5.91)] and telmisartan [WMD = −6.24 mmHg, (95% CI: −7.98 to −4.50)] demonstrated a more pronounced antihypertensive effect; however, due to the small number of relevant subgroup studies, these findings should be considered exploratory.

**TABLE 3:** SBP Regression-triple drug combinations.

Variables	Coefficient	95%CI	Std.Err	P value
Amlodipine Besylate + Olmesartan Medoxomil + Hydrochlorothiazide	0.33	(0.00,176.71)	1.00	0.718
Amlodipine + Aliskiren + Hydrochlorothiazide	0.52	(0.00,397.38)	1.66	0.840
Amlodipine + Azilsartan + Hydrochlorothiazide	14.57	(0.11,19,880.61)	50.56	0.449
Amlodipine + Losartan potassium + Chlorthalidone	1.45	(0.00,1286.86)	4.73	0.910
Amlodipine + Losartan + Hydrochlorothiazide	9.02	(0.15,5540.36)	27.84	0.484
Amlodipine + Olmesartan Medoxomil + Hydrochlorothiazide	18.18	(0.33,9987.07)	55.16	0.350
Amlodipine + Olmesartan + Hydrochlorothiazide	39.01	(0.09,16,995)	114.00	0.224
Amlodipine + Telmisartan + Hydrochlorothiazide	3.00	(0.00,2733.523)	9.83	0.741
Amlodipine + Valsartan + Hydrochlorothiazide	0.92	(0.00,454.10)	2.75	0.979

**TABLE 4:** SBP Regression-Baseline (SBP).

Variables	Coefficient	95%CI	Std.Err	P value
140–199 mmHg	0.11	(0.00,7.89)	0.22	0.294
160–179 mmHg	0.02	(0.00,1.70)	0.05	0.083
160–200 mmHg	0.03	(0.00,4.86)	0.08	0.172
<180 mmHg	2.08	(0.00,1184.11)	6.36	0.813
>140 mmHg	1.69	(0.02,151.82)	3.66	0.812
≥140 mmHg	0.02	(0.00,1.39)	0.04	0.069
≥145 mmHg	0.04	(0.00,2.98)	0.09	0.139
≥160 mmHg	4.25	(0.09,214.28)	8.05	0.451

**TABLE 5:** SBP Regression-Baseline (DBP).

Variables	Coefficient	95%CI	Std.Err	P value
100–109 mmHg	0.07	(0.00,31.16)	0.21	0.378
100–120 mmHg	0.07	(0.00,59.19)	0.23	0.425
90–109 mmHg	0.35	(0.00,272.84)	1.14	0.750
<110 mmHg	5.54	(0.00,13,012.18)	20.78	0.653
>90 mmHg	1.44	(0.00,849.59)	4.45	0.906
≥100 mmHg	2.19	(0.01,571.37)	5.89	0.773
≥95 mmHg	0.30	(0.00,111.48)	0.86	0.679

**TABLE 6:** SBP Regression-dual drug combinations.

Variables	Coefficient	95%CI	Std.Err	P value
Aliskiren + Hydrochlorothiazide	0.22	(8.56e-06,58.48)	0.82	0.318
Amlodipine Besylate + Hydrochlorothiazide	0.08	(0.00,2.72)	0.13	0.148
Amlodipine Besylate + Olmesartan Medoxomil	1.49	(0.045,49.20)	2.43	0.810
Amlodipine + Aliskiren	2.77	(0.03,227.31)	5.69	0.628
Amlodipine + Azilsartan	20.06	(0.24,1706.25)	41.56	0.170
Amlodipine + Chlorthalidone	22.20	(0.00,308,886)	98.7439	0.497
Amlodipine + Hydrochlorothiazide	0.60	(0.02,17.08)	0.94	0.749
Amlodipine + Losartan	104.14	(2.97,3652.22)	172.73	0.014
Amlodipine + Losartan potassium	0.26	(0.01,13.38)	0.47	0.473
Amlodipine + Olmesartan	58.73	(3.09,1116.87)	80.65	0.010
Amlodipine + Olmesartan Medoxomil	221.93	(9.62,5122.40)	324.81	0.002
Amlodipine + Telmisartan	3.78	(0.13,110.68)	5.95	0.412
Amlodipine + Valsartan	4.06	(0.09,191.31)	7.29	0.449
Losartan potassium + Chlorthalidone	200.34	(0.02,1666482)	843.11	0.228
Losartan + Hydrochlorothiazide	0.14	(0.02,10.32)	0.27	0.339
Olmesartan Medoxomil + Hydrochlorothiazide	0.39	(0.01,10.65)	0.60	0.551

**TABLE 7:** SBP Regression-treatment duration.

Variables	Coefficient	95%CI	Std.Err	P value
8 weeks	0.00	(0.00,0.10)	0.01	0.001
10 weeks	0.22	(0.01,3.99)	0.31	0.290
12 weeks	0.01	(0.00,0.12)	0.01	0.001

### DBP

**FIGURE 8:**
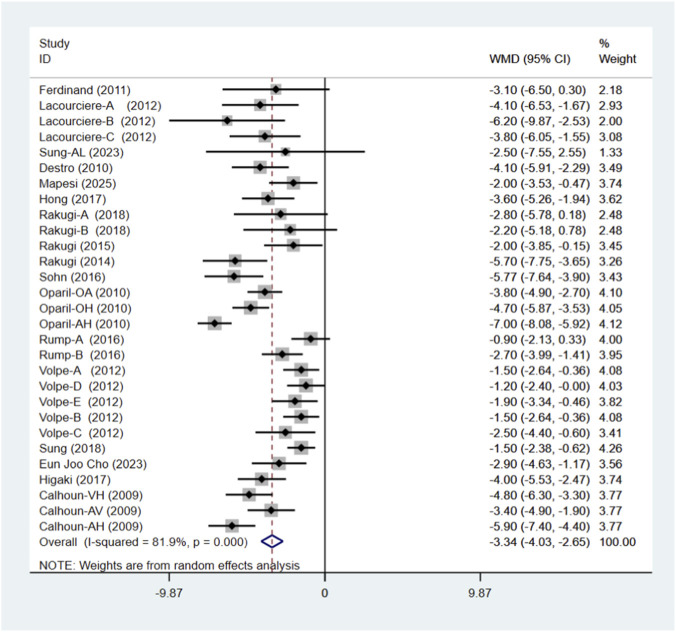
DBP forest plot.

**FIGURE 9:**
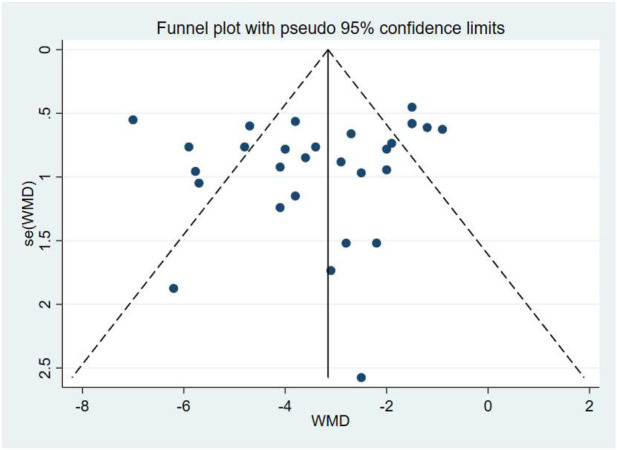
DBP - funnel graph.

**FIGURE 10:**
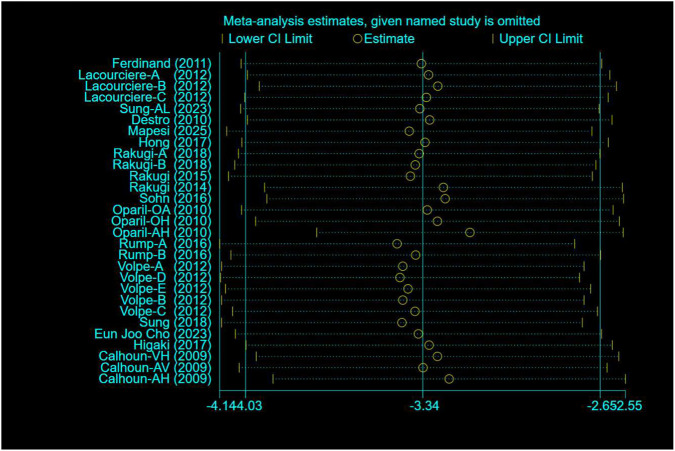
DBP - sensitivity analysis.

**FIGURE 11:**
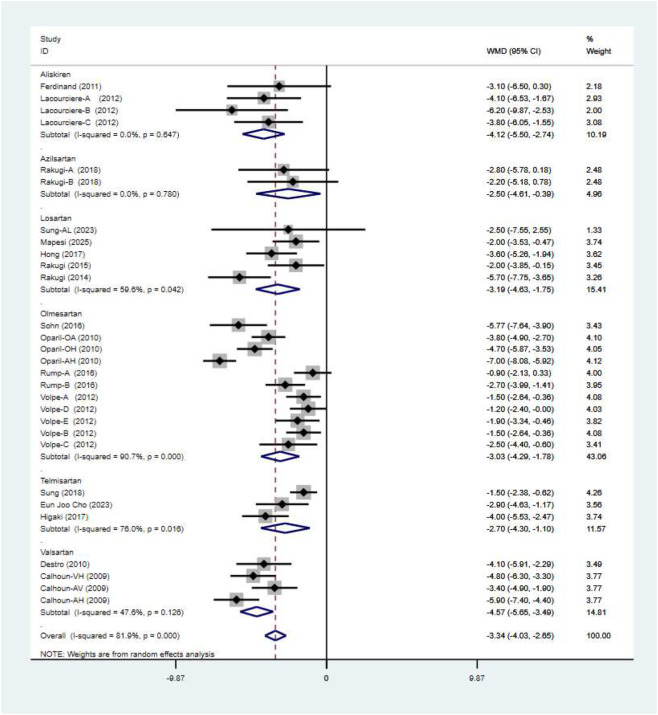
DBP subgroup.

A total of 17 studies comprising 29 randomised controlled trials were included, comparing the blood pressure-lowering effects of triple versus dual antihypertensive therapy on diastolic blood pressure. Due to the high level of heterogeneity in the study (I^2^ = 81.9%, P = 0.000), a random-effects model was employed for the analysis. The results (Figure 8) demonstrated that triple therapy significantly outperformed dual therapy in reducing DBP [WMD = −3.34; (95%CI: 4.03,-2.65)]. The Egger test (P = 0.341) revealed a non-significant publication bias, and the funnel plot (Figure 9) exhibited overall symmetry, indicating no substantial publication bias. Due to considerable heterogeneity in SBP reduction, a sensitivity analysis was conducted by sequentially excluding studies. The results (see Figure 10) demonstrated robust stability of the findings, unaffected by individual studies. To further explore the potential sources of heterogeneity and assess the impact of different study characteristics on the blood pressure-lowering efficacy for diastolic blood pressure, a meta-regression analysis was performed. The results showed that the triple drug combinations (Table 8), baseline systolic blood pressure (Table 9), and diastolic blood pressure (Table 10) all had no statistically significant effect on heterogeneity; among the dual drug combinations (Table 11), “Amlodipine + Losartan” (p = 0.034), “Amlodipine + Olmesartan” (P = 0.008), and “Amlodipine + Olmesartan Medoxomil” (P= 0.017) may be the causes of increased heterogeneity; treatment duration (Table 12)—8 weeks (P = 0.015) and 12 weeks (P = 0.001)—may also contribute to high heterogeneity. Further subgroup analysis by ARB type (Figure 11) showed that the blood pressure-lowering effects of different ARB triple-drug combinations varied. Triple combinations containing valsartan [WMD: −4.57 mmHg, (95% CI: −5.65, −3.49)] and losartan [WMD: −3.19 mmHg, (95% CI: −4.63, −1.75)] demonstrated a more pronounced effect on lowering diastolic blood pressure.

**TABLE 8:** DBP Regression - triple drug combinations.

Variables	Coefficient	95%CI	Std.Err	P value
Aliskiren + Amlodipine + Hydrochlorothiazide	0.19	(0.01,6.00)	0.31	0.324
Amlodipine Besylate + Olmesartan Medoxomil + Hydrochlorothiazide	0.07	(0.00,1.82)	0.11	0.104
Amlodipine + Losartan potassium + Chlorthalidone	0.42	(0.01,21.74)	0.79	0.650
Amlodipine + Losartan + Hydrochlorothiazide	0.54	(0.02,16.10)	0.88	0.710
Amlodipine + Olmesartan Medoxomil + Hydrochlorothiazide	0.66	(0.24,18.42)	1.05	0.798
Amlodipine + Olmesartan + Hydrochlorothiazide	2.28	(0.10,52.53)	3.42	0.589
Amlodipine + Telmisartan + Chlorthalidone	0.67	(0.01,45.59)	1.35	0.845
Amlodipine + Telmisartan + Hydrochlorothiazide	0.89	(0.03,29.12)	1.49	0.947
Amlodipine + Valsartan + Hydrochlorothiazide	0.13	(0.01,3.21)	0.20	0.197

**TABLE 9:** DBP regression - Baseline (SBP).

Variables	Coefficient	95%CI	Std.Err	P value
140–199 mmHg	0.78	(0.00,273.39)	2.18	0.929
160–179 mmHg	0.17	(0.00,61.89)	0.48	0.537
160–200 mmHg	0.15	(0.00,63.07)	0.43	0.518
>140 mmHg	0.61	(0.00,243.68)	1.74	0.864
≥140 mmHg	0.07	(0.00,24.30)	0.19	0.352
≥145 mmHg	0.11	(0.00,40.51)	0.31	0.446
≥150 mmHg	1.00	(0.00,542.59)	3.02	1.000
≥160 mmHg	2.23	(0.01,730.43)	6.20	0.775

**TABLE 10:** DBP regression - Baseline (DBP).

Variables	Coefficient	95%CI	Std.Err	P value
100–109 mmHg	0.18	(0.00,115.35)	0.55	0.582
100–120 mmHg	0.14	(0.00,107.61)	0.44	0.541
90–109 mmHg	0.28	(0.00,233.22)	0.89	0.695
90–114 mmHg	0.22	(0.00,278.77)	0.77	0.666
>90 mmHg	1.51	(0.00,1020.04)	4.74	0.896
≥100 mmHg	0.75	(0.00,403.35)	2.26	0.824
≥95 mmHg	0.21	(0.00,130.90)	0.65	0.620

**TABLE 11:** DBP Regression - dual drug combinations.

Variables	Coefficient	95%CI	Std.Err	P value
Aliskiren + Amlodipine	2.88	(0.13,63.84)	4.16	0.476
Aliskiren + Hydrochlorothiazide	0.25	(0.00,26.13)	0.54	0.530
Amlodipine Besylate + Hydrochlorothiazide	0.11	(0.01,1.55)	0.14	0.096
Amlodipine + Hydrochlorothiazide	1.00	(0.09,11.31)	1.13	0.997
Amlodipine + Losartan	16.44	(1.28,211.94)	19.60	0.034
Amlodipine + Losartan potassium	3.83	(0.22,67.92)	5.13	0.334
Azilsartan + Amlodipine	9.98	(0.41,241.65)	14.82	0.144
Losartan + Hydrochlorothiazide	0.41	(0.02,10.59)	0.62	0.563
Olmesartan Medoxomil + Amlodipine	20.65	(1.86,229.58)	23.19	0.017
Olmesartan Medoxomil + Amlodipine Besylate	2.72	(0.19,38.56)	3.36	0.432
Olmesartan Medoxomil + Hydrochlorothiazide	0.76	(0.64,9.00)	0.87	0.813
Olmesartan + Amlodipine	23.85	(2.65,215.17)	24.46	0.008
Telmisartan + Amlodipine	9.82	(0.98,98.20)	10.55	0.052
Valsartan + Amlodipine	4.06	(0.23,71.79)	5.43	0.314

**TABLE 12:** DBP Regression - treatment duration.

Variables	Coefficient	95%CI	Std.Err	P value
8 weeks	0.16	(0.04,0.68)	0.11	0.015
12 weeks	0.07	(0.02,0.29)	0.05	0.001
16 weeks	1.02	(0.12,8.79)	1.06	0.987

### Blood pressure control rates

**FIGURE 12:**
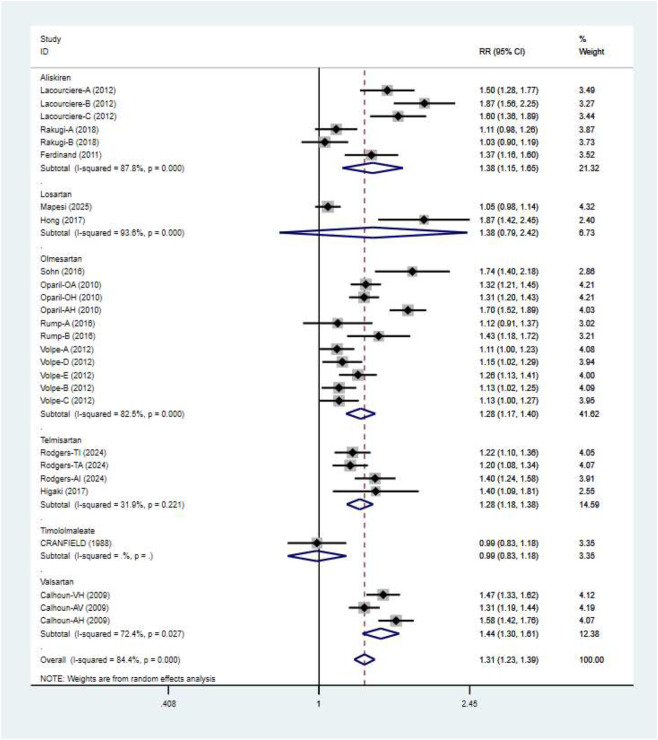
Blood pressure control rates - subgroup.

**FIGURE 13:**
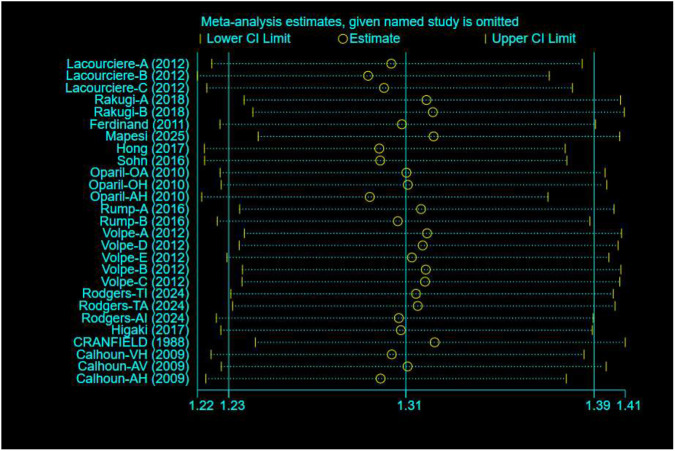
Blood pressure control rates-Sensitivity analysis.

**FIGURE 14:**
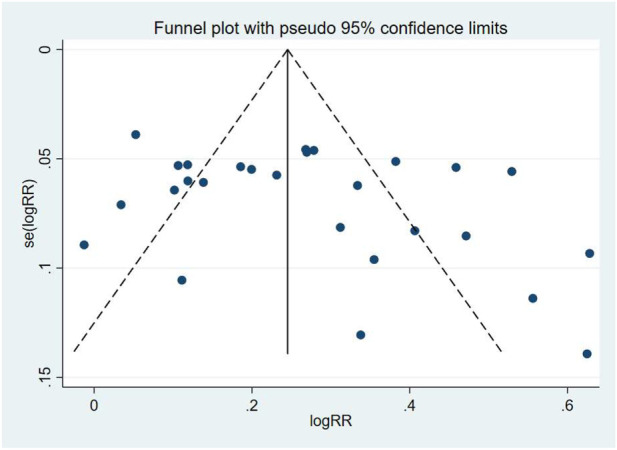
Blood pressure control rates-Funnel Graph.

Blood pressure control rates were analyzed using a cutoff of systolic/diastolic blood pressure <140/90 mmHg. Triple therapy significantly improved blood pressure control rates [RR = 1.31; (95%CI:1.23,1.39)]. Subgroup analysis (Figure 12) indicated that olmesartan [RR = 1.28, (95%CI:1.17,1.40)], telmisartan [RR = 1.28, (95%CI:1.18,1.38)], and valsartan [RR = 1.44, (95%CI:1.30,1.61)] significantly increased control rates. However, high heterogeneity existed among studies (I^2^ = 84.4%). Sensitivity analysis (Figure 13) indicated robust pooled effect sizes, with the overall RR consistently ranging between 1.22 and 1.41 after excluding individual studies. Funnel plots (Figure 14) exhibited essentially symmetrical patterns, and the Egger test did not suggest significant publication bias (P = 0.102). To further explore the potential sources of heterogeneity and assess the impact of different study characteristics on the blood pressure-lowering efficacy, a meta-regression analysis was conducted. The results showed that neither the triple drug combinations (Table 13) nor the duration of treatment (Table 14) had a statistically significant effect on heterogeneity; among the dual drug combinations (Table 15), “Amlodipine + Losartan” (p= 0.003), “Amlodipine + Olmesartan” (P = 0.007), “Azilsartan + Amlodipine” (P= 0.005), and “Spironolactone + Propranolol hydrochloride” (P= 0.009) may be the causes of increased heterogeneity; Baseline systolic blood pressure (Table 16) in the ranges “160–179 mmHg” (P = 0.006), “160–200 mmHg” (P = 0.002), and “≥140 mmHg” (P= 0.023) may be contributing factors to the high heterogeneity. Baseline diastolic blood pressure (Table 17) in the ranges “>90” (P= 0.015), “≥100” (P = 0.016), and “≥95” (P = 0.019) may be contributing factors to high heterogeneity.

**TABLE 13:** Blood pressure control rates Regression - triple drug combinations.

Variables	Coefficient	95%CI	Std.Err	P value
Amiloride Hydrochloride + Timololmaleate + Hydrochlorothiazide	0.70	(0.41,1.20)	0.18	0.185
Amlodipine Besylate + Olmesartan Medoxomil + Hydrochlorothiazide	1.02	(0.65,1.59)	0.21	0.933
Amlodipine + Aliskiren + Hydrochlorothiazide	0.98	(0.64,1.50)	0.20	0.905
Amlodipine + Losartan potassium + Chlorthalidone	1.33	(0.74,2.39)	0.37	0.315
Amlodipine + Losartan + Hydrochlorothiazide	0.75	(0.45,1.25)	0.18	0.251
Amlodipine + Olmesartan medoxomil + Hydrochlorothiazide	1.00	(0.63,1.58)	0.22	0.996
Olmesartan + Amlodipine + Hydrochlorothiazide	0.82	(0.53,1.26)	0.17	0.351
Telmisartan + Rmlodipine + Indapamide	0.91	(0.58,0.41)	0.19	0.643
Valsartan + Hydrochlorothiazide + Amlodipine	1.03	(0.66,1.61)	0.22	0.884

**TABLE 14:** Blood pressure control rates Regression - treatment duration.

Variables	Coefficient	95%CI	Std.Err	P value
8 weeks	1.28	(1.00,1.65)	0.16	0.053
10 weeks	0.89	(0.70,1.13)	0.10	0.327
12 weeks	1.04	(0.82,1.31)	0.12	0.746

**TABLE 15:** Blood pressure control rates Regression - dual drug combinations.

Variables	Coefficient	95%CI	Std.Err	P value
Aliskiren + Amlodipine	0.98	(0.79,1.20)	0.09	0.805
Aliskiren + Hydrochlorothiazide	1.28	(0.97,1.69)	0.16	0.080
Amlodipine Besylate + Hydrochlorothiazide	1.16	(0.94,1.43)	0.10	0.149
Amlodipine + Hydrochlorothiazide	1.08	(0.90,1.31)	0.09	0.366
Amlodipine + Indapamide	0.95	(0.76,1.19)	0.09	0.635
Amlodipine + Losartan	0.72	(0.60,0.86)	0.06	0.003
Amlodipine + Losartan potassium	1.27	(0.87,1.87)	0.22	0.188
Azilsartan + Amlodipine	0.73	(0.60,0.89)	0.06	0.005
Olmesartan Medoxomil + Amlodipine	0.87	(0.69,1.10)	0.09	0.215
Olmesartan Medoxomil + Amlodipine Besylate	0.90	(0.74,1.10)	0.08	0.261
Olmesartan Medoxomil + Hydrochlorothiazide	0.94	(0.78,1.23)	0.08	0.447
Olmesartan + Amlodipine	0.79	(0.67,0.92)	0.06	0.007
Spironolactone + Propranolol hydrochloride	0.67	(0.51,0.89)	0.08	0.009
Telmisartan + Amlodipine	0.84	(0.69,1.02)	0.07	0.081
Telmisartan + Indapamide	0.83	(0.68,1.03)	0.08	0.079
Valsartan + Amlodipine	0.89	(0.73,1.09)	0.08	0.228

**TABLE 16:** Blood pressure control rates Regression - Baseline (SBP).

Variables	Coefficient	95%CI	Std.Err	P value
140–179 mmHg	1.18	(0.93,1.51)	0.14	0.163
160–179 mmHg	1.49	(1.14,1.95)	0.19	0.006
160–200 mmHg	1.54	(1.19,1.99)	0.19	0.002
>140 mmHg	1.08	(0.81,1.42)	0.14	0.593
≥140 mmHg	1.33	(1.05,1.69)	0.15	0.023
≥145 mmHg	1.25	(0.99,1.58)	0.14	0.056
≥160 mmHg	1.10	(0.88,1.37)	0.11	0.378

**TABLE 17:** Blood pressure control rates Regression - Baseline (DBP).

Variables	Coefficient	95%CI	Std.Err	P value
100–109 mmHg	0.98	(0.73,1.30)	0.13	0.862
90–114 mmHg	0.85	(0.55,1.31)	0.18	0.449
>90 mmHg	0.73	(0.57,0.94)	0.09	0.015
≥100 mmHg	0.76	(0.61,0.94)	0.08	0.016
≥95 mmHg	0.75	(0.60,0.95)	0.08	0.019

**FIGURE 15:**
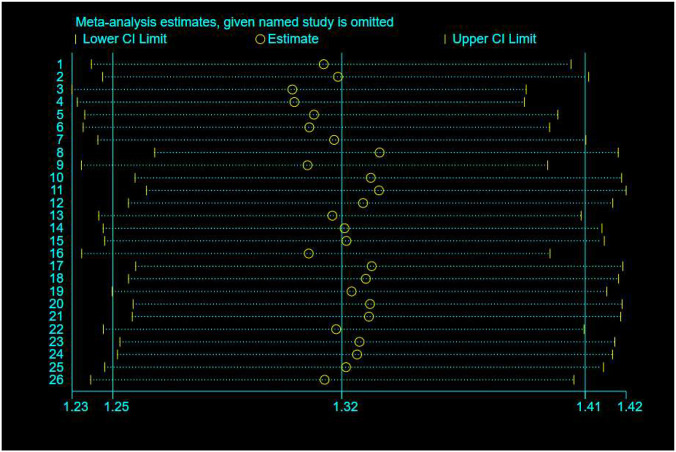
Blood pressure control rates (excluding the 1988 study)-sensitivity analysis.

**FIGURE 16:**
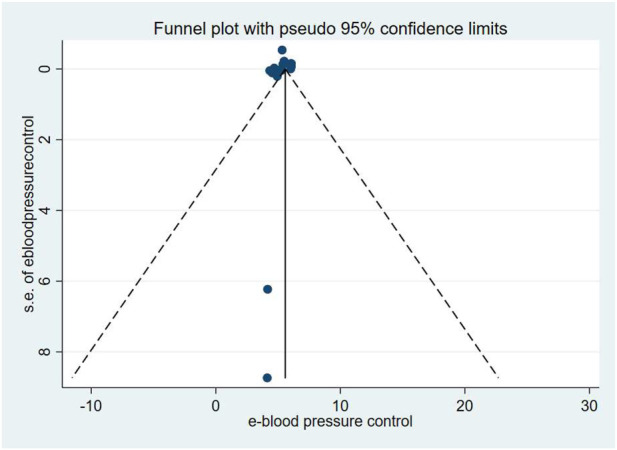
Blood pressure control rates (excluding the 1988 study)-Funnel Graph.

Given the wide time span of the studies (1988–2025), there may be significant differences from existing research regarding blood pressure targets, diagnostic criteria, background care, adverse event reporting standards, and drug formulations. Therefore, after excluding the 1988 study, we conducted a sensitivity analysis (Figure 15), and the overall RR remained consistently between 1.25 and 1.41. The funnel plot (Figure 16) exhibits an asymmetrical shape, and the Egger test suggests the presence of publication bias (P = 0.047). After applying the Trim and Fill method, publication bias was still detected (P = 0.000).

### Adverse events

Regarding safety, this study evaluated 14 adverse reaction symptoms—including headache, cough, hypotension, hypokalemia, dizziness, upper respiratory tract infection, fatigue, and peripheral edema—associated with treatment using the triple-drug regimen versus the dual-drug regimen. The results showed (Table 18) that triple therapy improved fatigue in patients [RR: 0.80, (95% CI: 0.67, 0.96)]; for constipation [RR: 0.88; (95% CI: 0.22, 3.50)], cough [RR: 0.93; (95% CI: 0.63, 1.38)], nasopharyngitis [RR: 1.10; (95% CI: 0.86, 1.41)], and upper respiratory symptoms [RR: 0.80; (95% CI: 0.62, 1.04)], peripheral edema [RR: 1.05; (95% CI: 0.79, 1.39)], nausea [RR: 1.18; (95% CI: 0.93, 1.50)], headache [RR: 1.08; (95% CI: 0.95, 1.23)]; and hypokalemia [RR: 0.62; (95% CI: 0.23, 1.68)]. elevated serum uric acid [RR: 6.34, (95% CI: 2.42, 16.63)], hypotension [RR: 3.10, (95% CI: 1.71, 5.61)], muscle cramps [RR: 1.56, (95% CI: 1.13, 2.13)], dizziness [RR: 1.53, (95% CI: 1.35, 1.74)], and dyspepsia [RR: 2.53, (95% CI: 1.39, 4.58)]. A subgroup analysis of the adverse reactions associated with increased risk revealed that the risk of elevated serum uric acid was primarily concentrated in triple-drug regimens containing telmisartan [RR = 7.16, (95%CI: 2.17, 23.60)] and losartan [RR = 12.37, (95%CI: 1.62, 94.52)]. This adverse reaction was primarily observed in four trials across three studies; one study (Hong et al., 2017) demonstrated that this symptom was unrelated to the medication, while two studies (Higaki et al., 2017; Rakugi et al., 2018) attributed it to diuretics. Hypotension was primarily caused by triple-drug regimens containing olmesartan [RR: 3.41; (95%CI: 1.70, 6.82)]. Muscle tremors [RR: 1.97; (95% CI: 1.14, 3.40)] and dyspepsia [RR: 2.45; (95%CI: 1.32, 4.56)] were primarily caused by triple-drug regimens containing valsartan. Dizziness was observed in various drug subgroups, including those containing olmesartan [RR: 1.49, (95%CI: 1.27, 1.74)], valsartan [RR: 1.71, 95%CI: 1.29, 2.29], and telmisartan [RR: 3.85, 95% CI: 1.09, 8.77] showed a statistically significant increase in risk. There were no statistically significant differences among subgroups (P= 0.138), but moderate heterogeneity was observed overall (I^2^ = 36.8%, P = 0.025).

**TABLE 18:** Adverse event.

Adverse event	Any grade			
	Study	Heterogeneity		RR (95%CI)
		P	I^2^(%)	
Blood uric acid	4	0.452	0.0	6.34 (2.42,16.63)
Constipation	3	0.438	0.0	0.88 (0.22,3.50)
Cough	12	0.320	12.7	0.93 (0.63,1.38)
Fatigue	15	0.686	0.0	0.80 (0.67,0.96)
Nasopharyngitis	14	0.813	0.0	1.10 (0.86,1.41)
Dizziness	30	0.025	36.6	1.53 (1.35,1.74)
Hypotension	15	0.884	0.0	3.10 (1.71,5.61)
Hypokalemia	7	0.005	67.6	0.62 (0.23,1.68)
Upper respiratory	11	0.879	0.0	0.80 (0.62,1.04)
Peripheral edema	24	0.000	75.9	1.05 (0.79,1.39)
Nausea	14	0.786	0.0	1.18 (0.93,1.50)
Dyspepsia	5	0365	7.3	2.53 (1.39,4.58)
Headache	33	0.351	7.1	1.08 (0.95,1.23)
Muscle spasms	7	0.956	0.0	1.56 (1.13,2.13)

CI, confidence interval.

### Publication bias

The risk of bias assessment for included studies indicated that most studies were rated as risk of “unclear bias” regarding random sequence generation and allocation concealment, primarily due to insufficient methodological descriptions in the original literature. Regarding performance bias and measurement bias, the vast majority of studies were rated as “low risk” suggesting that the measurement of primary efficacy outcomes was minimally influenced by subjective factors. Selective reporting bias posed a low overall risk across studies, indicating good data integrity. Under “other biases”no study registered high risk in any domain. Furthermore, visual funnel plot analysis for primary systolic and diastolic blood pressure outcomes revealed no significant asymmetry, and Egger’s tests showed no statistical significance (P = 0.431; P = 0.341), indicating no substantial publication bias.Although some studies reported insufficiently on randomization and allocation concealment methods, the key blinding design and outcome completeness were good, and no significant publication bias was detected.

## Discussion

This study updates previous meta-analyses. Compared with the analysis by Salam et al. (2019), which included 14 studies and 11,457 patients, this study extended the search period to July 2025 and ultimately included 21 studies and 17,669 patients, further demonstrating that triple therapy is more effective than dual therapy in lowering SBP and DBP and improving blood pressure control rates. Overall, these results support the notion that adding a third class of antihypertensive medication provides additional short-term blood pressure-lowering benefits in patients with inadequate control on dual therapy. Wang et al. (2025) suggested that there are significant differences in blood pressure management among different drug classes. Unlike previous studies that focused on “whether to add a third drug,” this study further conducted a subgroup analysis of the ARB class within the triple therapy regimens. This is because different ARBs exhibit variations in pharmacokinetic and pharmacodynamic characteristics, which theoretically may influence blood pressure-lowering efficacy and tolerability; simultaneously, since the included studies predominantly employed the fixed combination of “CCB + ARB + thiazide diuretic” in their triple-drug regimens, the ARB subclass emerged as a clinically relevant stratification factor. It should be emphasized that the comparisons of different ARB classes in this study are derived from subgroup analyses at the study level and constitute indirect and exploratory evidence. Although some regimens demonstrated greater blood pressure reductions numerically, this does not prove that any particular ARB is superior to others in terms of long-term clinical outcomes. Therefore, these results are better suited as clues for generating hypotheses rather than being interpreted as clinical recommendations for the preferential use of specific ARBs.

Triple-drug therapy effectively reduces blood pressure, which is consistent with the findings of Abdullah and Asim (2025) and O'Hagan et al. (2023). Significant heterogeneity was observed in the analyses of both SBP and DBP in this study. Through meta-regression, we found that some of this heterogeneity may be related to differences in dual-drug baseline regimens and treatment duration, rather than being entirely attributable to the drug composition of the triple-drug intervention itself or baseline blood pressure. However, these supplementary analyses can only partially explain the differences among studies; other unmeasured clinical and methodological factors may still influence the effect estimates. Therefore, although the main results are generally consistent in direction and sensitivity analyses indicate that the overall conclusions are reasonably robust, the magnitude of the effect should still be interpreted with caution. In the ARB subgroup analysis of this study, triple therapy regimens containing valsartan demonstrated a greater reduction in blood pressure. This finding may be related to valsartan’s receptor affinity, tissue penetration, half-life, and metabolic pathways; combination therapy containing valsartan may be more effective in maintaining 24-h blood pressure stability (Müller and Stegbauer, 2026; Kario et al., 2023). It is important to emphasize that these subgroup analyses are exploratory in nature, based on indirect cross-study comparisons, and do not establish that any particular ARB is superior to others in terms of long-term clinical outcomes. Therefore, the relevant results should be regarded as exploratory findings rather than definitive evidence to guide clinical practice. Regarding the outcome of blood pressure control rates, the high heterogeneity may also be related to the wide time span of the studies, as well as differences in blood pressure control targets and background treatment standards across different periods. Even after excluding early studies, publication bias persisted, suggesting that the pooled results for this outcome should be interpreted with caution.

In terms of safety, this study provides a more detailed summary than previous reviews, incorporating a variety of specific adverse reactions rather than limiting the analysis to any adverse events or treatment discontinuation due to adverse events. The results indicate that the overall tolerability of triple therapy is acceptable, but the risk of certain specific adverse reactions may be increased, and differences may exist between different treatment regimens. It should be noted that this study did not uniformly redefine the definitions of adverse events across studies but instead used data from the original research reports. Furthermore, the analysis of certain specific adverse reactions was based on a limited number of studies, resulting in insufficient precision in the estimates and involving multiple comparisons. For example, the risk of hyperuricemia associated with triple therapy primarily stems from diuretics rather than ARBs themselves. If a patient has already developed a significant elevation in serum uric acid, consideration may be given to substituting or co-administering an ARB with a mild uricosuric effect within the triple therapy regimen to partially offset the uric acid-raising effect of diuretics (Horio et al., 2024). Therefore, the association between drug class and specific adverse reactions should be regarded as an exploratory finding and requires further research for validation.

From a clinical perspective, current guidelines support the use of combination antihypertensive therapy in patients with poorly controlled blood pressure; however, the timing of initiating triple therapy and the specific drug regimen should still be determined based on a comprehensive assessment of patient risk stratification, tolerability, and treatment goals. Previous meta-analyses have shown (Blood Pressure Lowering Treatment Trialists, 2021) that for every 5 mmHg reduction in systolic blood pressure, the risk of major cardiovascular events decreases by 10%, with even more significant reductions in the risk of stroke and heart failure. Most of the trial participants included in this study were patients who had not achieved target blood pressure levels despite dual therapy or who had high baseline blood pressure. Our results suggest that in this population, adding a third class of medication may be more effective for improving short-term blood pressure control than simply increasing the dose. However, the primary endpoints of this study—changes in blood pressure, rate of blood pressure control, and short-term adverse events—are all surrogate endpoints. Because most of the included trials had short follow-up periods and there is a lack of long-term randomized evidence with primary endpoints such as cardiovascular events, stroke, heart failure, or all-cause mortality, this study cannot yet demonstrate that triple therapy is superior to dual therapy in terms of clinical outcomes. This gap in evidence also limits the possibility of drawing stronger clinical inferences regarding specific triple-therapy strategies.

In addition, triple therapy can be administered in various forms in clinical practice, including free combination, two-tablet regimens, or single-pill fixed-dose combinations (SPCs). Currently, the World Health Organization (WHO) has included fixed-dose combination (SPC) antihypertensive medications in the 2019 Essential Medicines List (EML) to promote their use and improve hypertension control. Existing studies suggest that SPCs may help improve adherence and offer certain long-term management benefits (Weisser et al., 2020; Borghi and Granados, 2023; Marschner et al., 2025; Masi et al., 2024). However, the relative advantages of different dosing regimens in terms of efficacy, adherence, treatment inertia, and health economics still require further evaluation in the context of specific populations and clinical settings.

Although this study included a larger number of studies and patients, conducted comprehensive subgroup analyses and sensitivity tests, and performed detailed assessments of various adverse reactions as well as risk stratification by ARB class, we hope to enhance the clinical applicability of the results. However, this study still has certain limitations: First, there were differences among the included studies in terms of patients’ baseline blood pressure, drug regimens, and treatment duration, leading to high heterogeneity in some results. Although meta-regression analysis was performed, it could not fully explain the sources of heterogeneity; therefore, caution should be exercised when interpreting the combined effects. Second, most included studies were short-term randomized controlled trials with treatment durations concentrated between 8 and 12 weeks. The lack of long-term data exceeding 6 months makes it difficult to assess the true advantages of triple therapy regarding long-term blood pressure control stability, target organ protection, and cardiovascular event endpoints; Third, this study did not include clinical outcomes such as cardiovascular death, myocardial infarction, stroke, and hospitalization for heart failure, limiting the ability to draw direct inferences in clinical practice; Fourth, in the safety analysis, the number of studies supporting certain adverse reactions was small, with wide confidence intervals, and the certainty of evidence varied across different outcomes, suggesting that estimates of these effects require further validation in future studies. Additionally, factors such as patient medication adherence, quality of life, and economic costs were not assessed during the study.

## Conclusion

Compared with dual-drug regimens, triple-drug antihypertensive regimens may be more effective for short-term blood pressure control, and their overall safety profile is acceptable. However, the subgroup analyses in this study represent exploratory findings only and cannot be directly used to guide clinical drug selection. Given that the evidence for the primary efficacy outcome is of moderate certainty but still exhibits significant heterogeneity, the results should be interpreted with caution. Future studies of high quality, with long-term follow-up and a focus on clinical outcomes, are needed to verify the long-term benefits and risks of triple therapy.
