# Supplementary material for: Comparative efficacy and safety of dual-combination vs. triple-combination antihypertensive therapies in hypertensive patients: an updated meta-analysis of randomized controlled trials
Source: Front Pharmacol. 2026 May 15;17:1786728. doi: 10.3389/fphar.2026.1786728 (PMC13219965; doi:10.3389/fphar.2026.1786728)
Supplement: Supplementary file 1 [file Supplementaryfile1.docx]

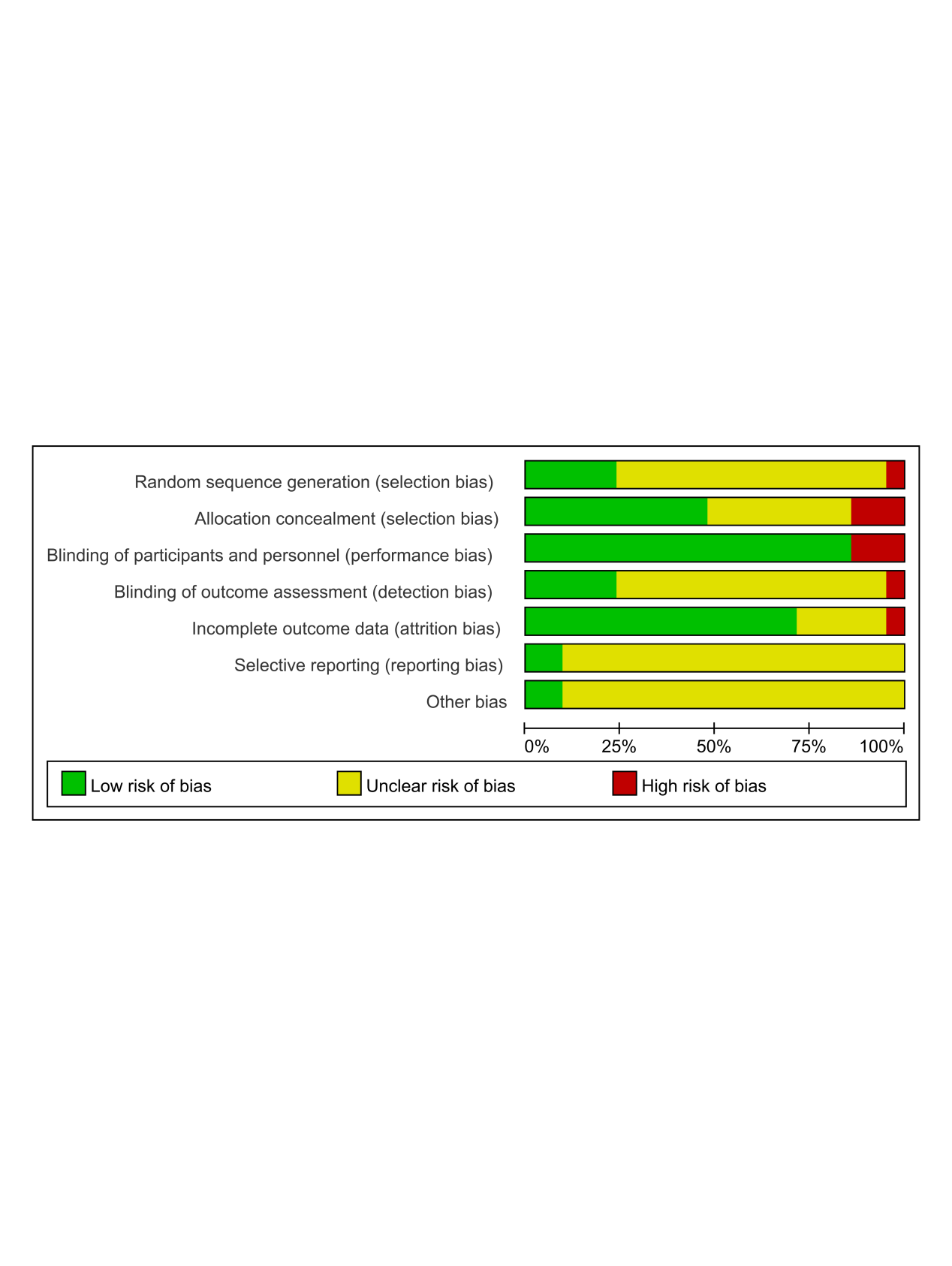


Figures 2:Risk of bias graph


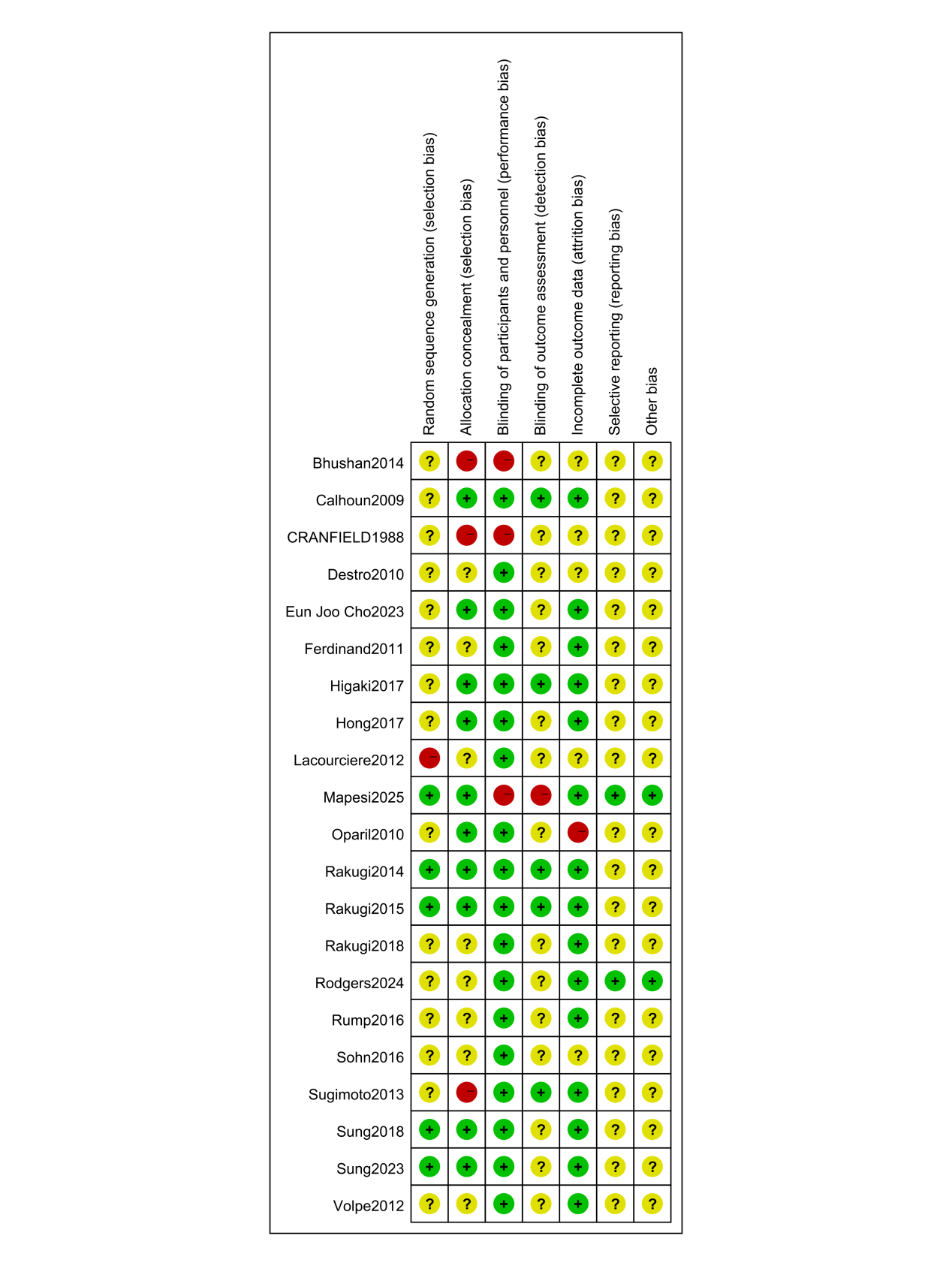


Figure 3 : Risk bias of summary


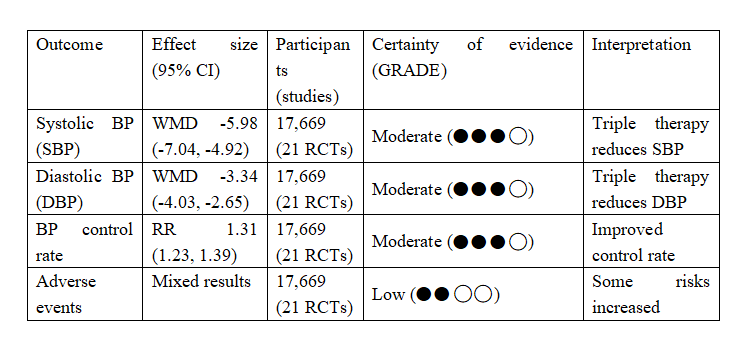


Table 2：GRADE


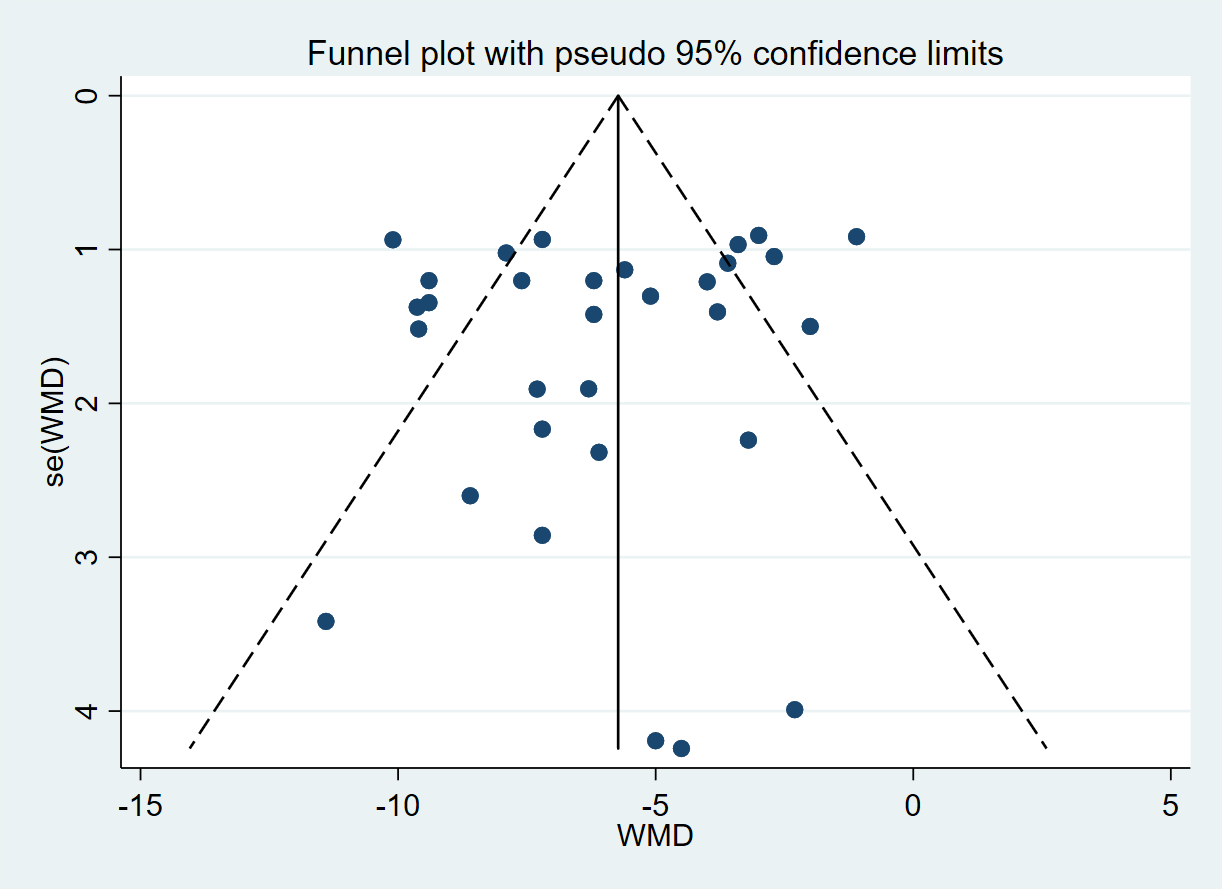


Figure 5:SBP Funnel Graph


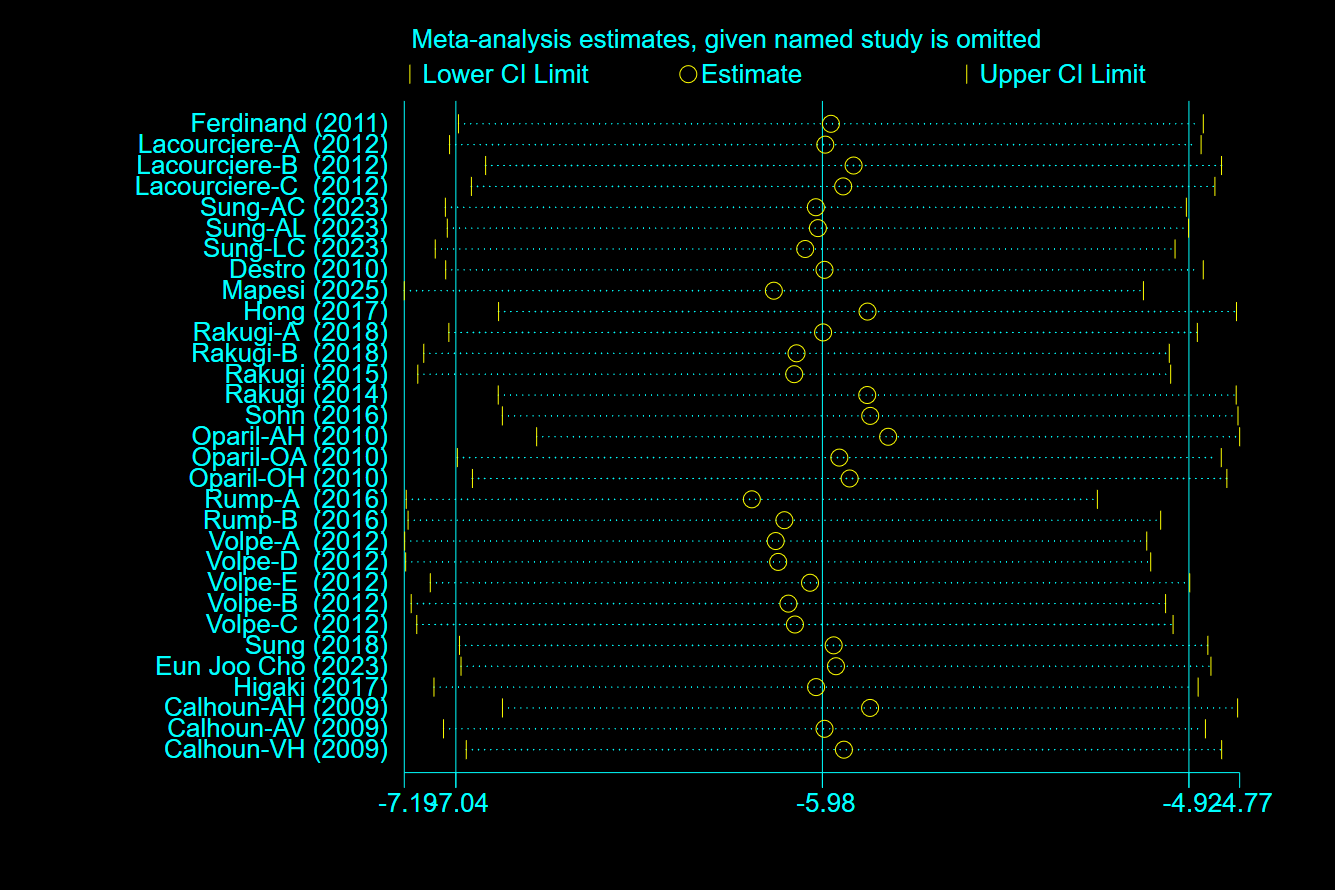


Figure 6:SBP sensitivity analysis


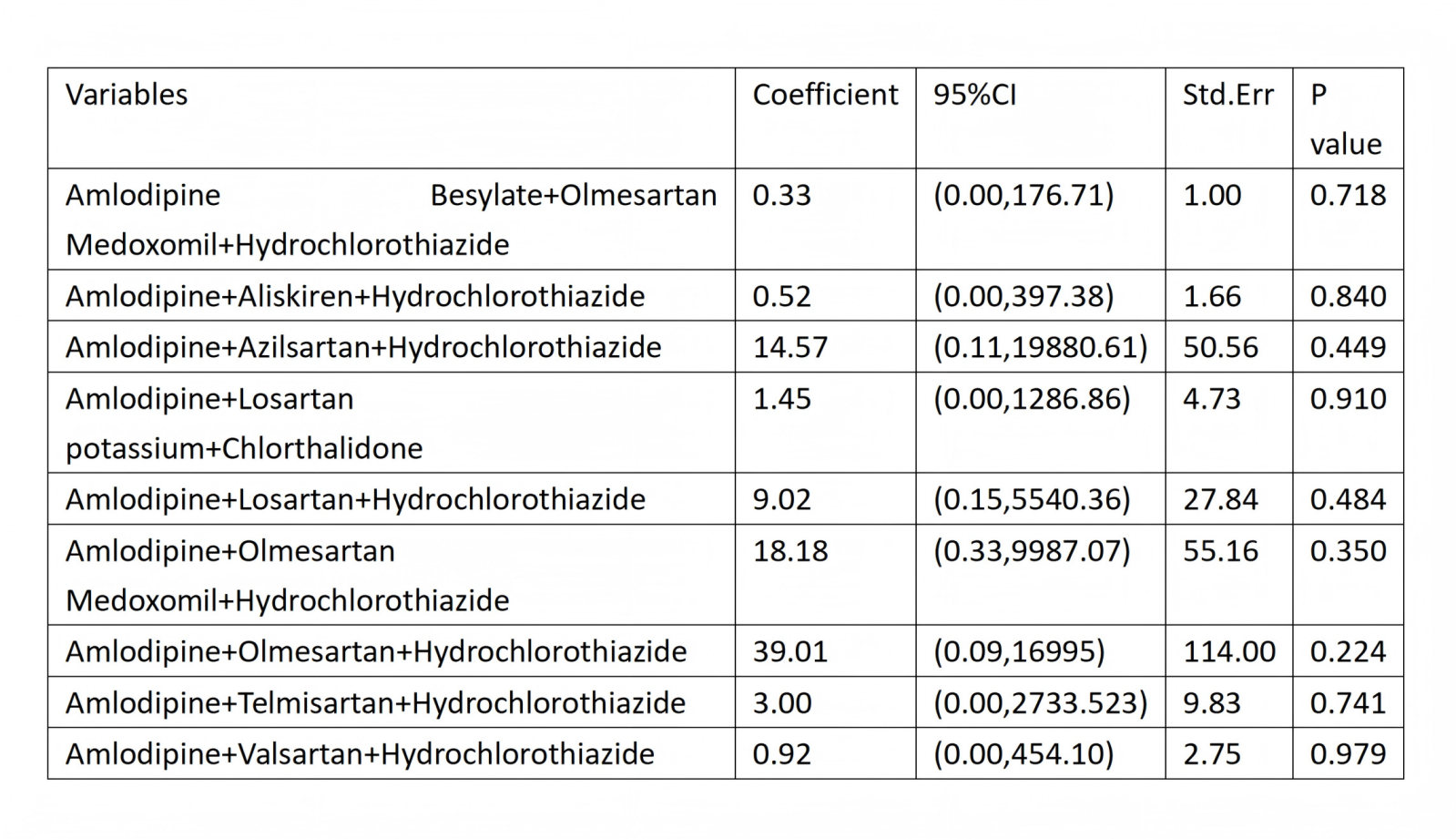


Table 3 :SBP Regression-triple drug combinations


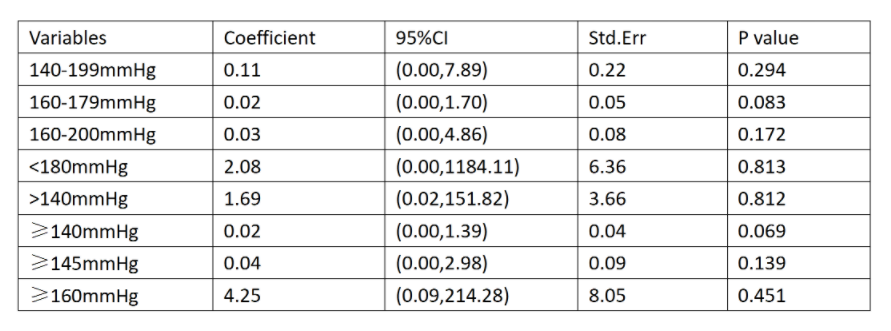


Table 4:SBP Regression-Baseline(SBP)


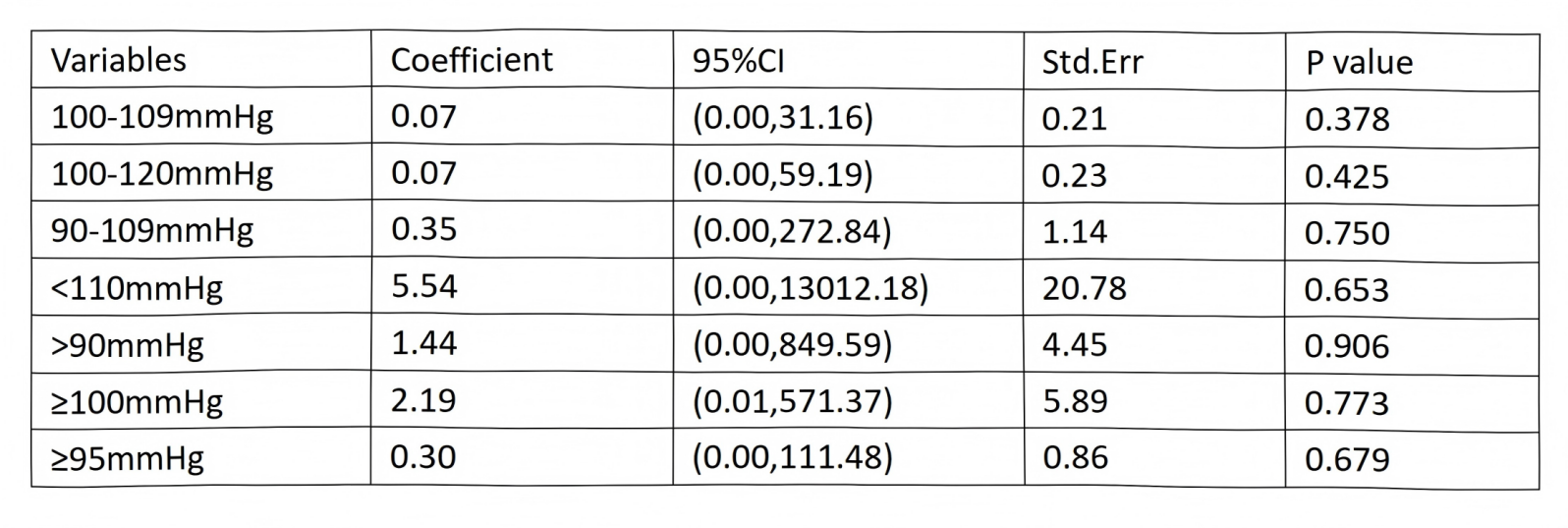


Table 5:SBP Regression-Baseline(DBP)


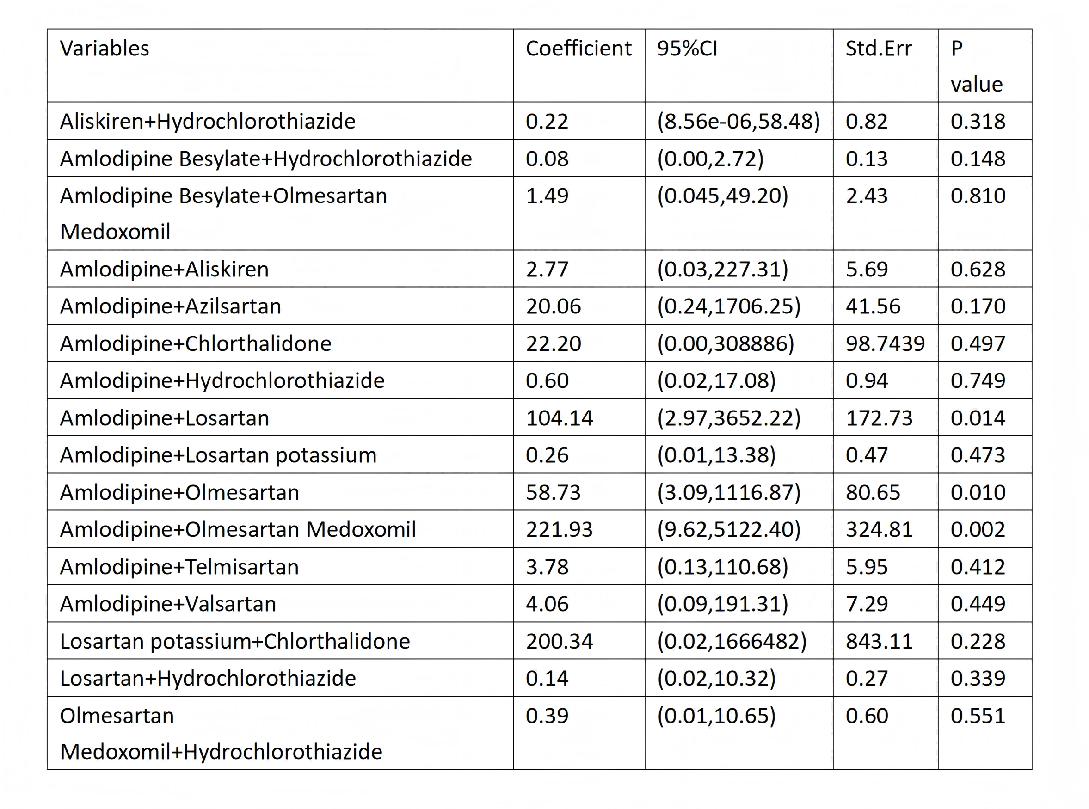


Table 6:SBP Regression-dual drug combinations


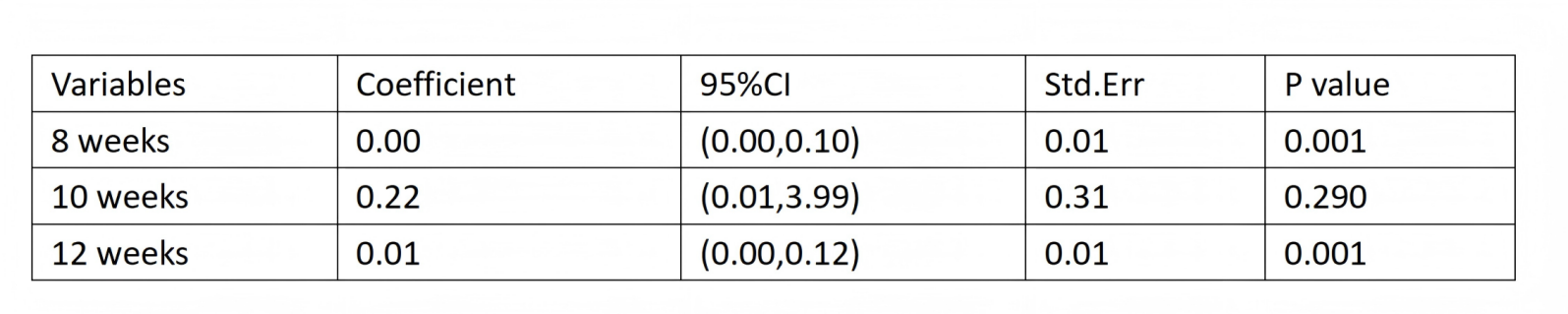


Table 7:SBP Regression- treatment duration


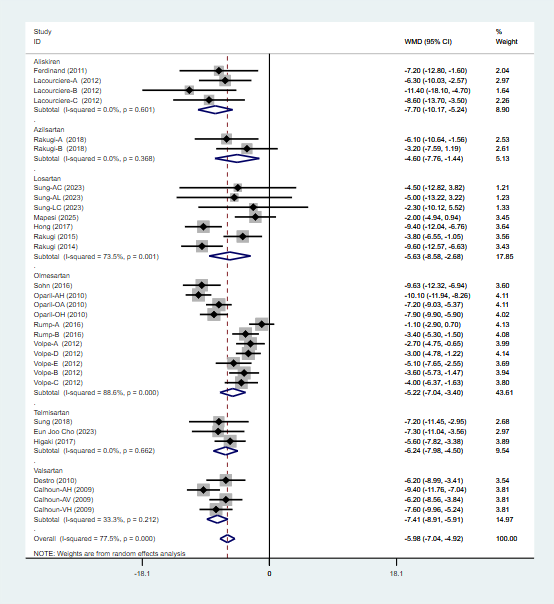


Figure 7:SBP-subgroups


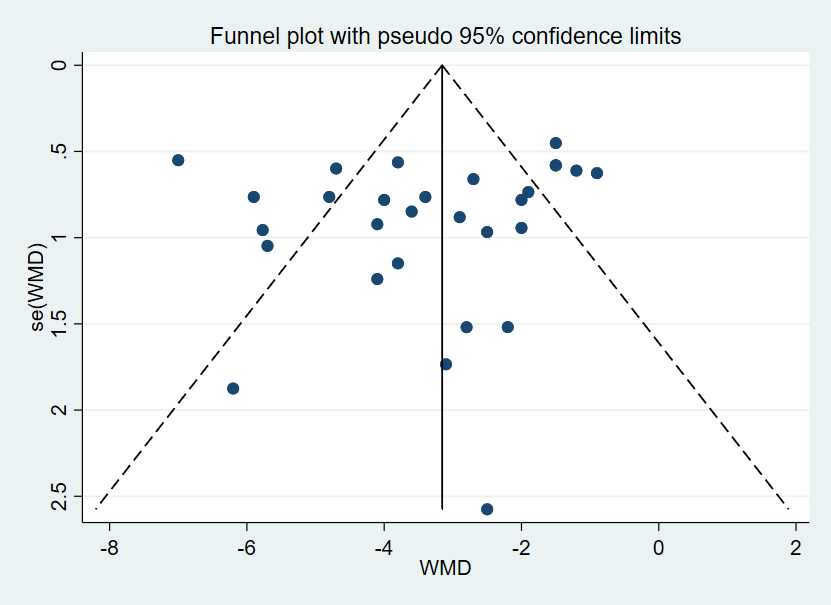


Figure 9:DBP - Funnel Graph


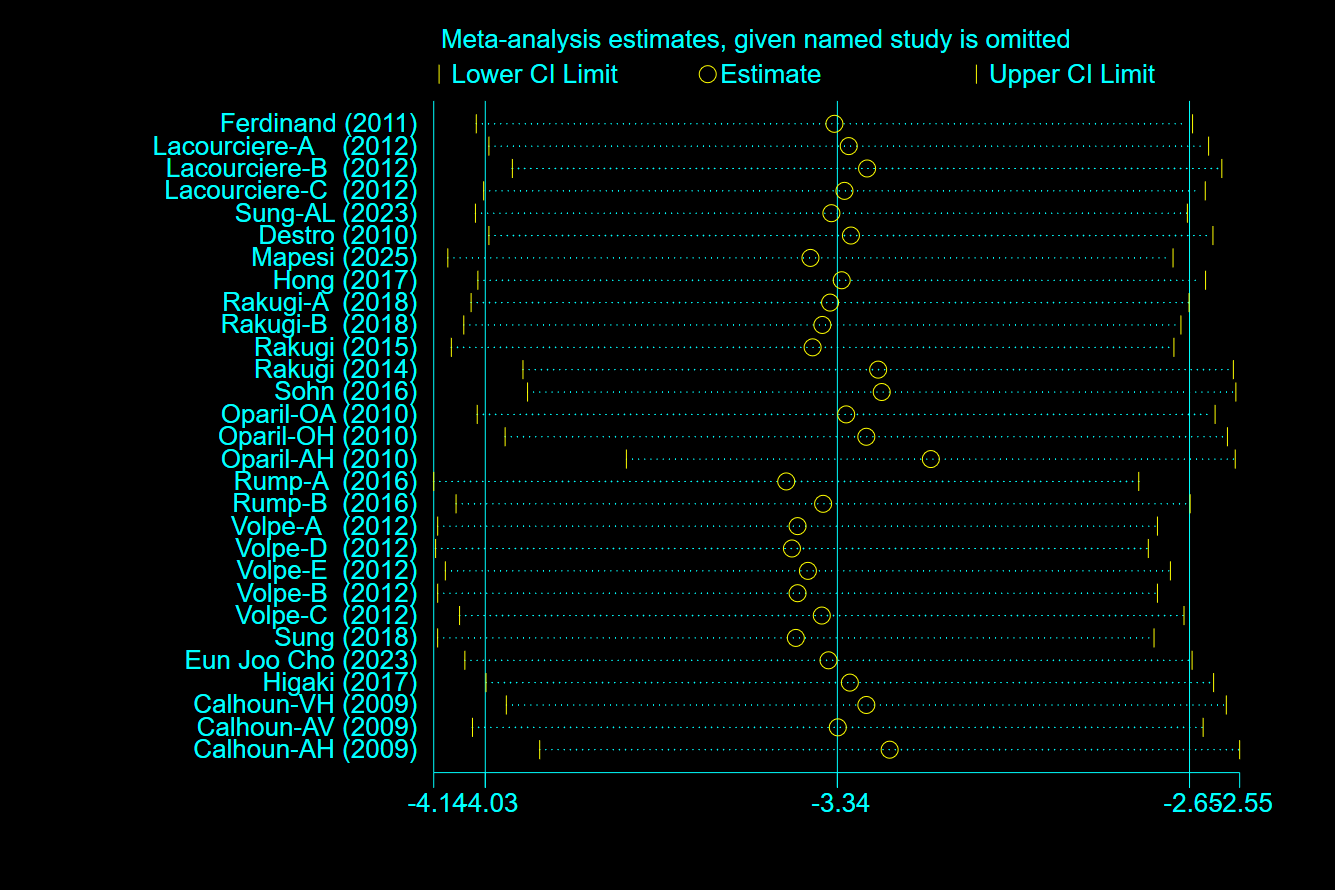


Figure 10: DBP - sensitivity analysis


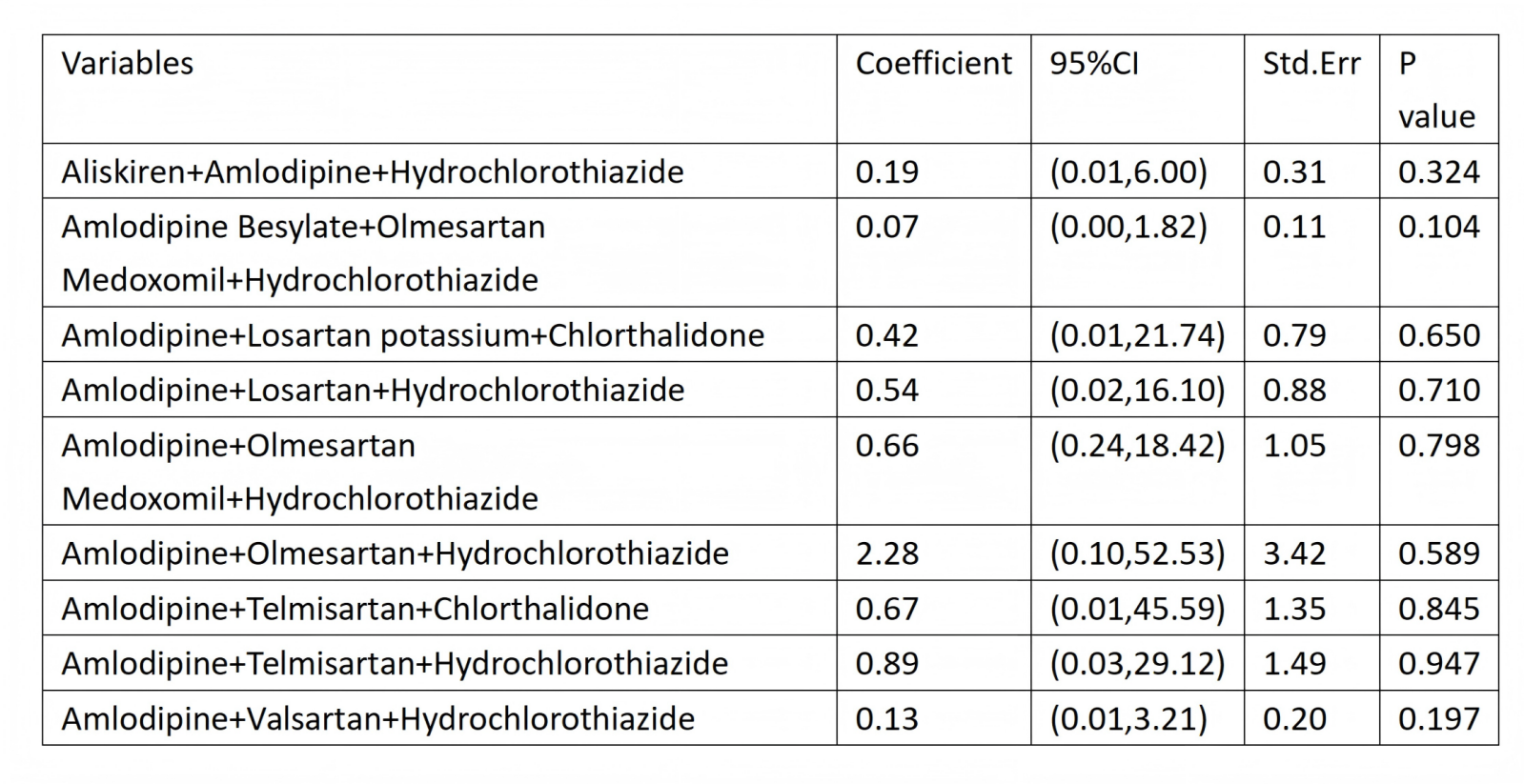


Table 8:DBP Regression - triple drug combinations


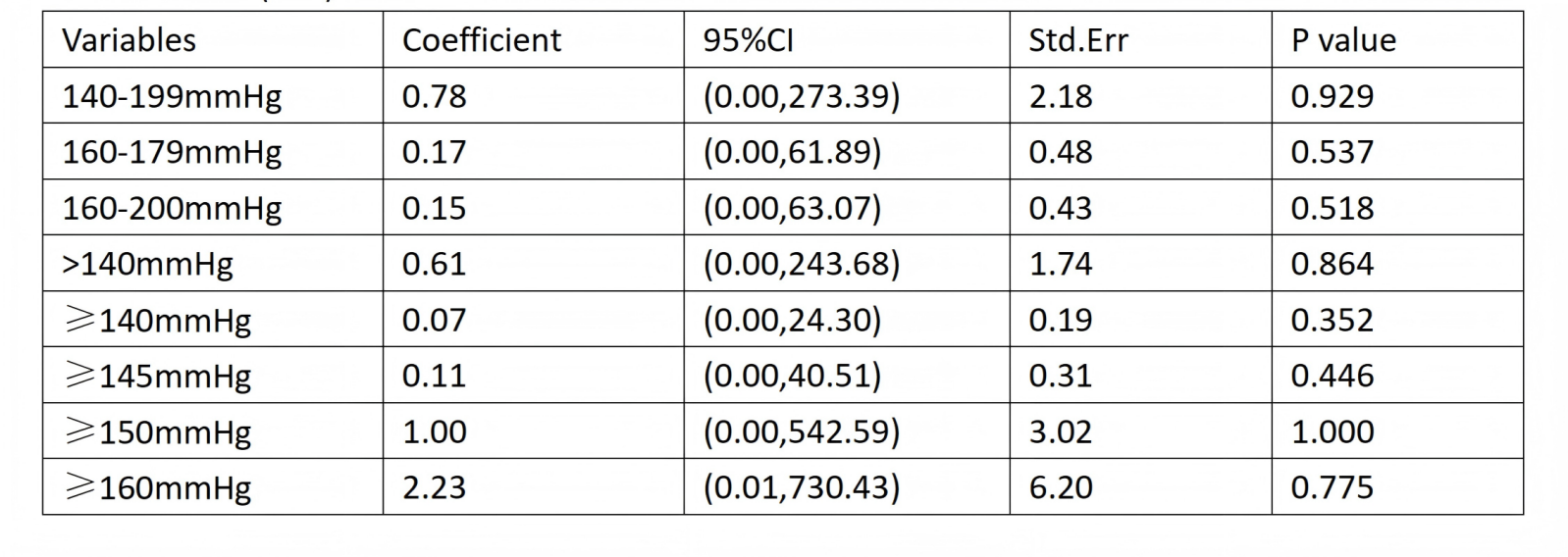


Table 9:DBP Regression - Baseline(SBP)


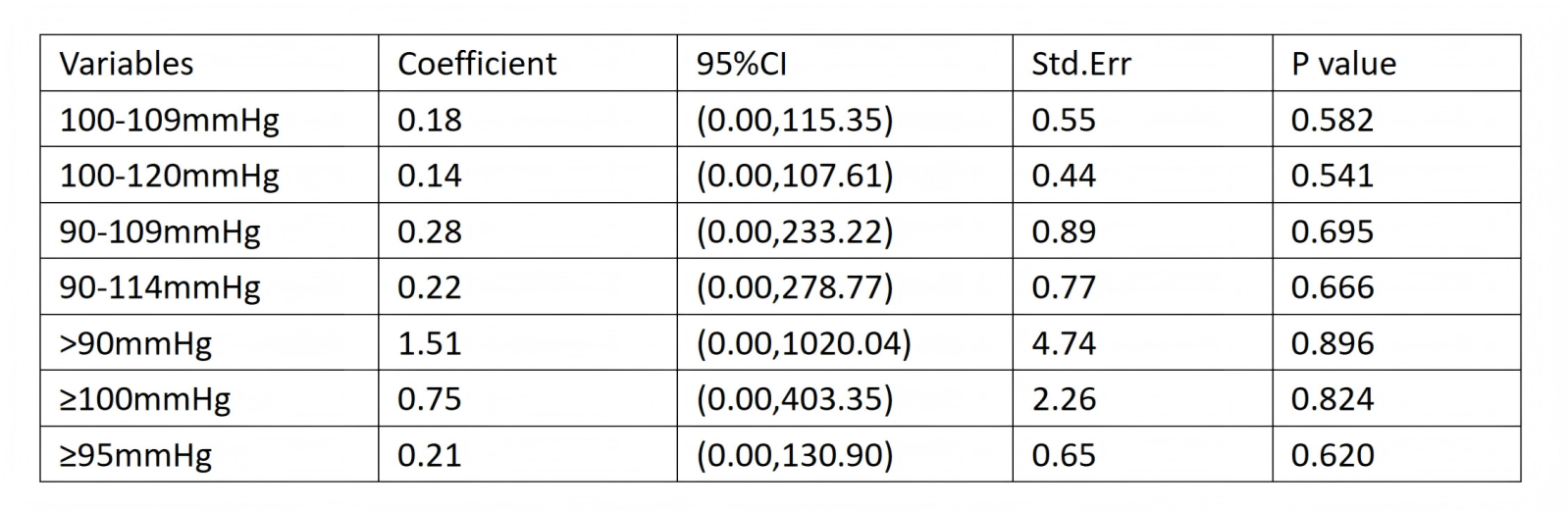


Table 10:DBP Regression - Baseline(DBP)


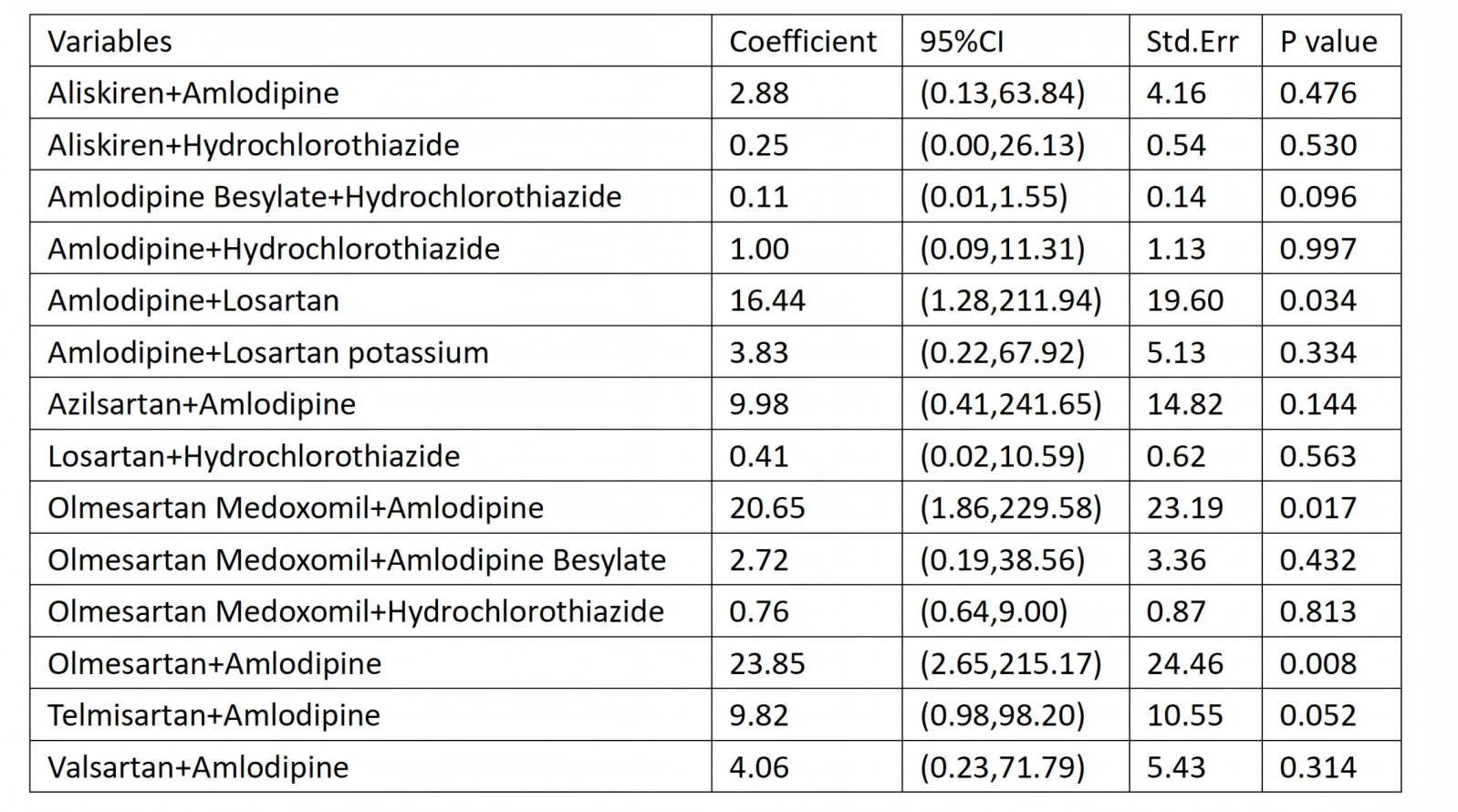


Table 11:DBP Regression - dual drug combinations


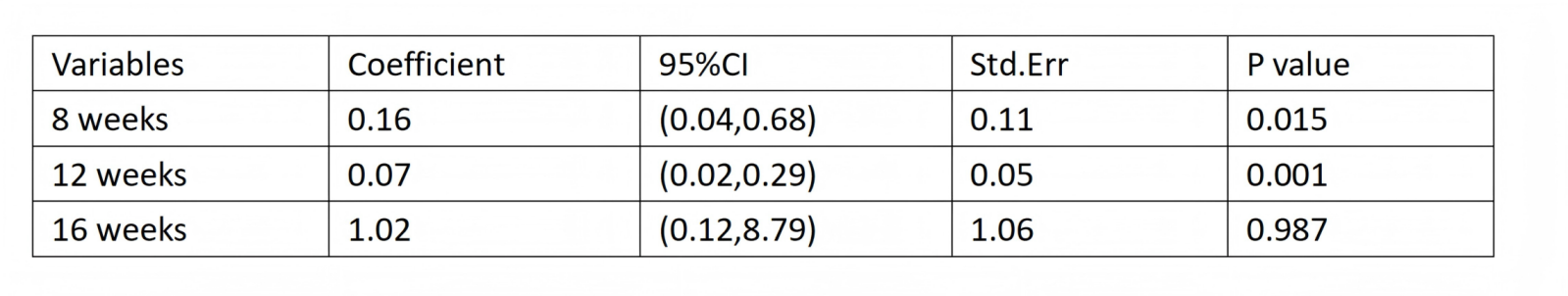


Table 12:DBP Regression - treatment duration


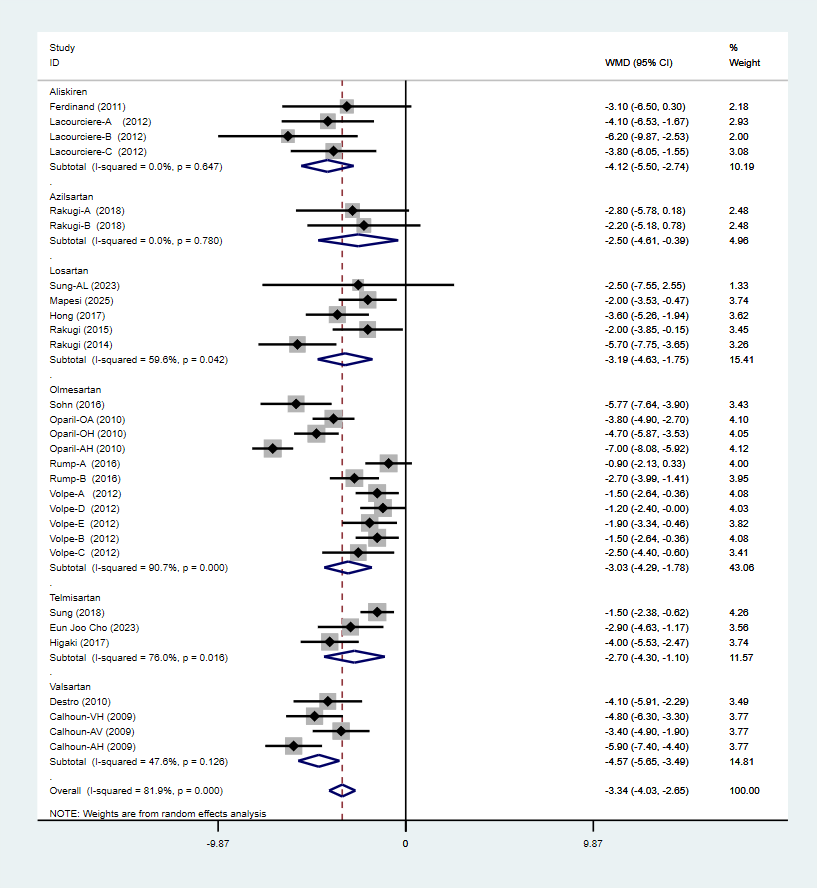


Figure 11 DBP subgroup


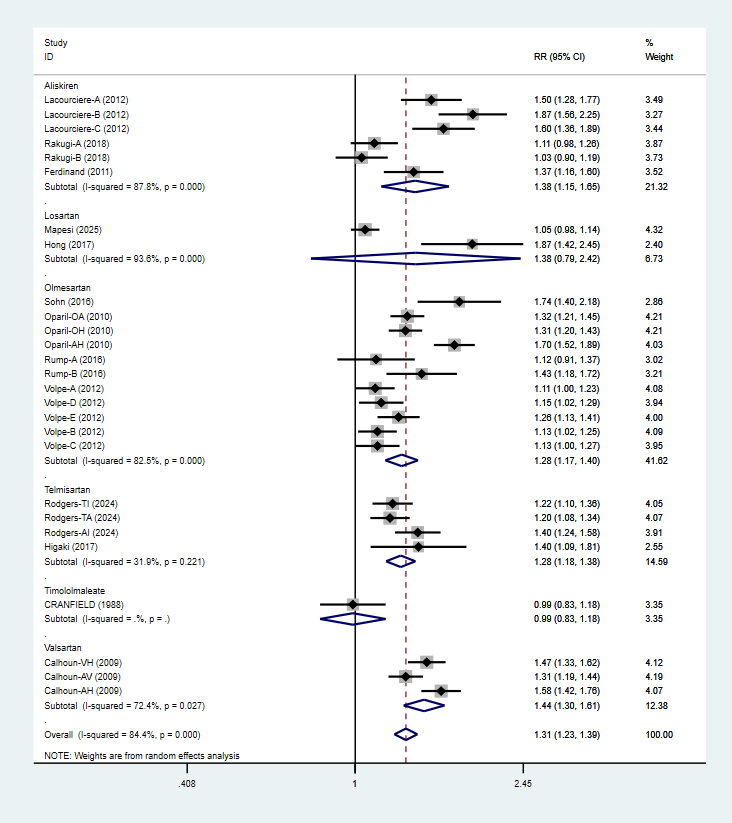


Figure 12 Blood Pressure Control Rates - Subgroup


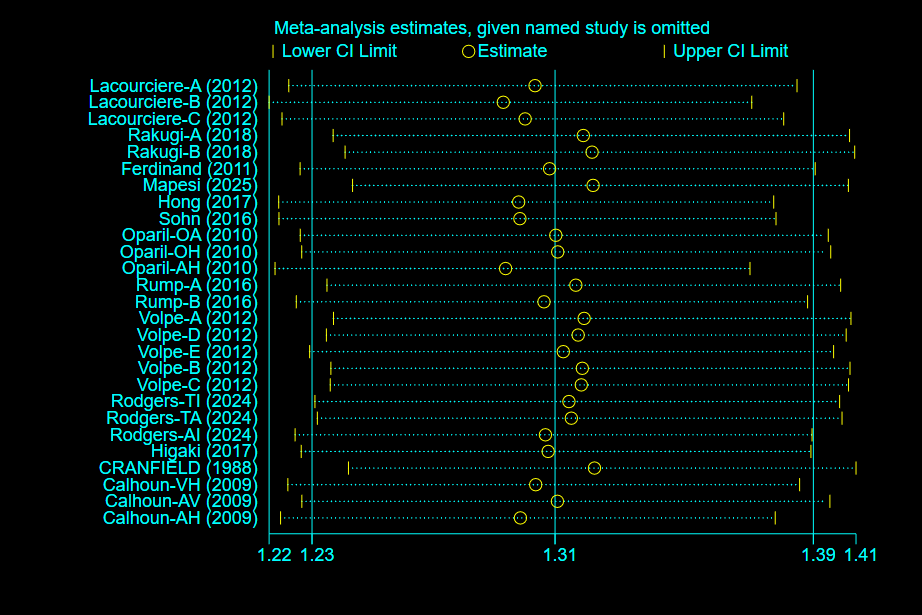


Figure 13:Blood pressure control rates-Sensitivity analysis


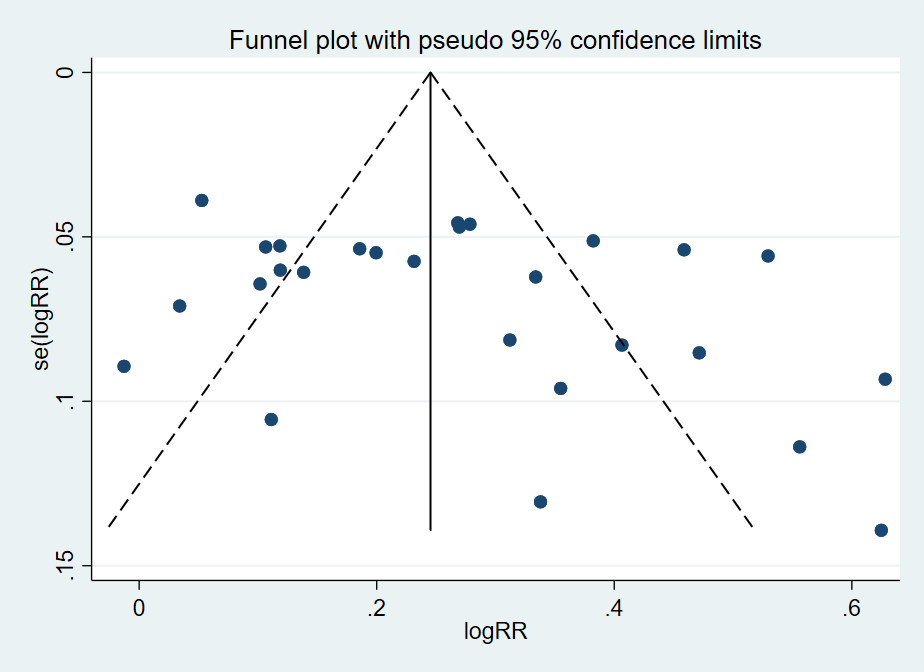


Figure 14:Blood pressure control rates-Funnel Graph


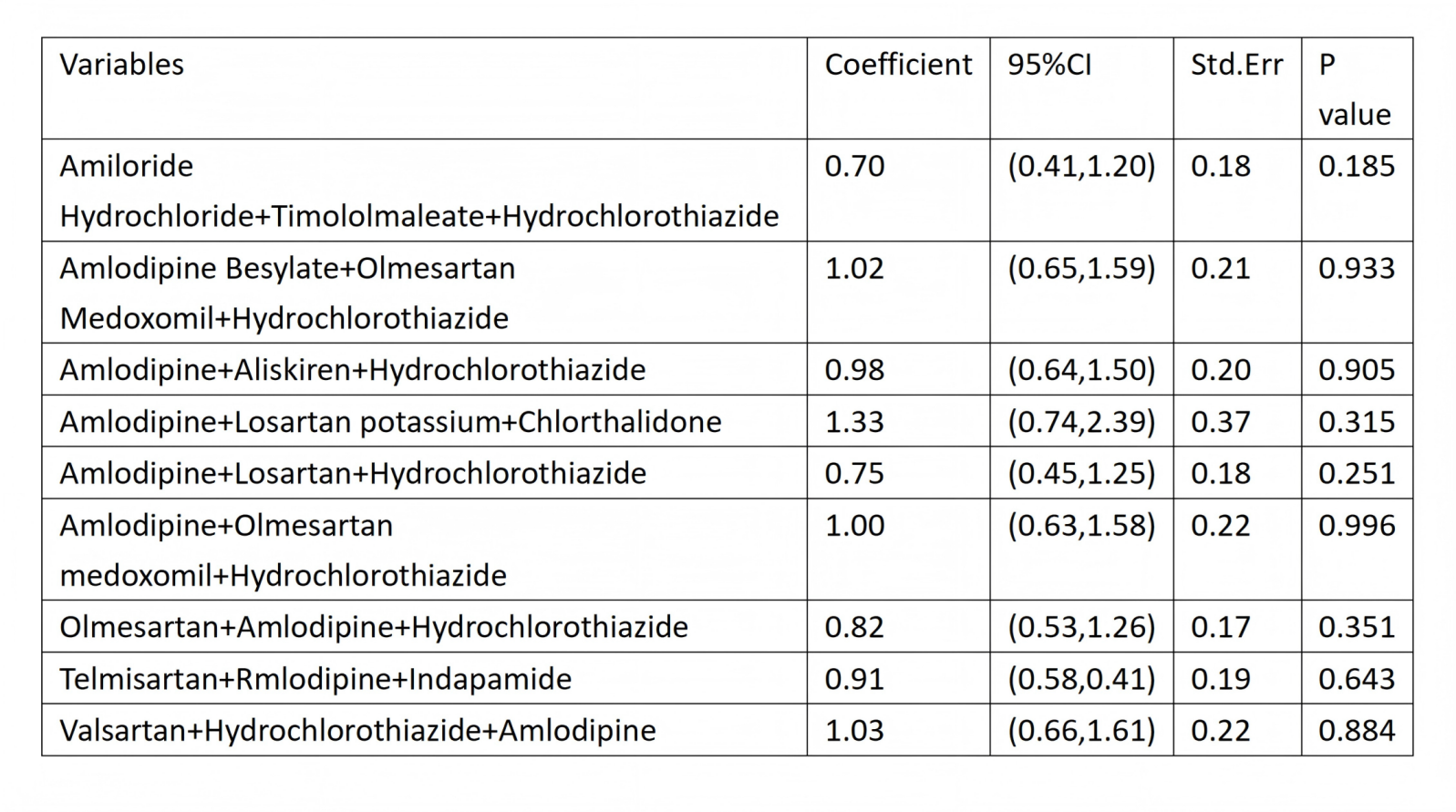


Table 13:Blood pressure control rates Regression - triple drug combinations


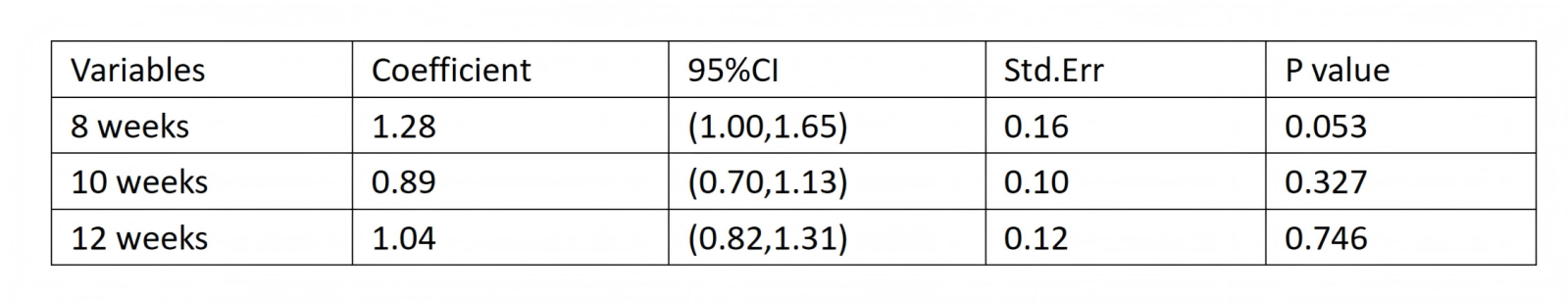


Table14:Blood pressure control rates Regression - treatment duration


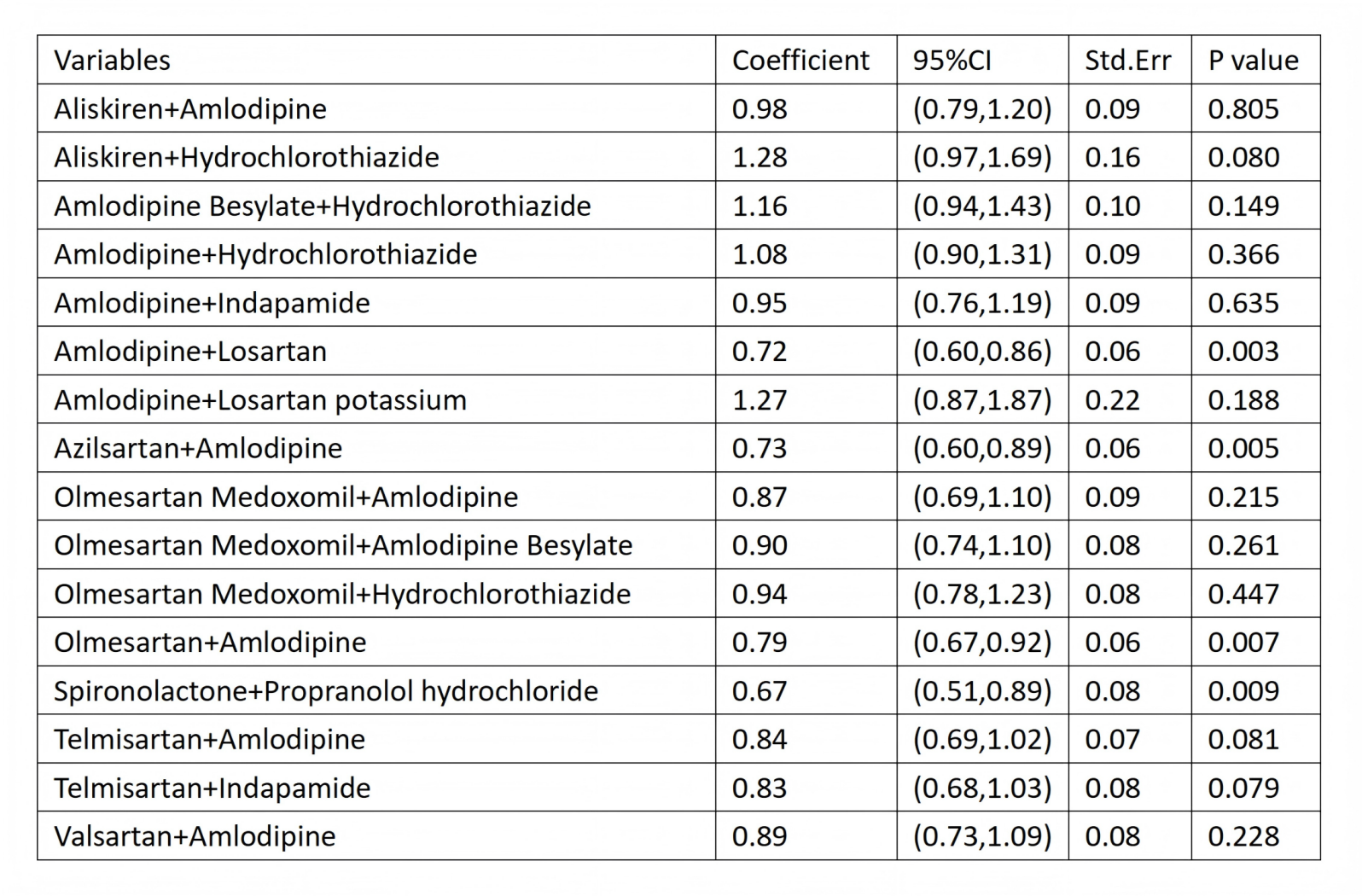


Table15:Blood pressure control rates Regression - dual drug combinations


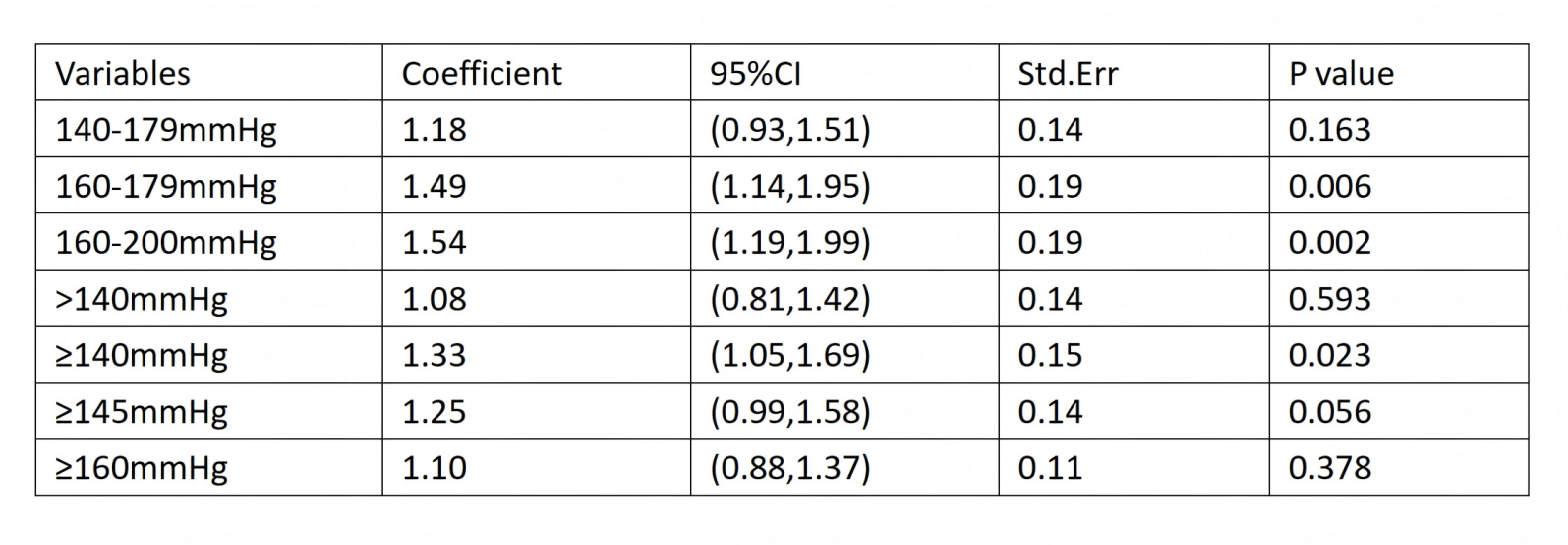


Table16:Blood pressure control rates Regression - Baseline(SBP)


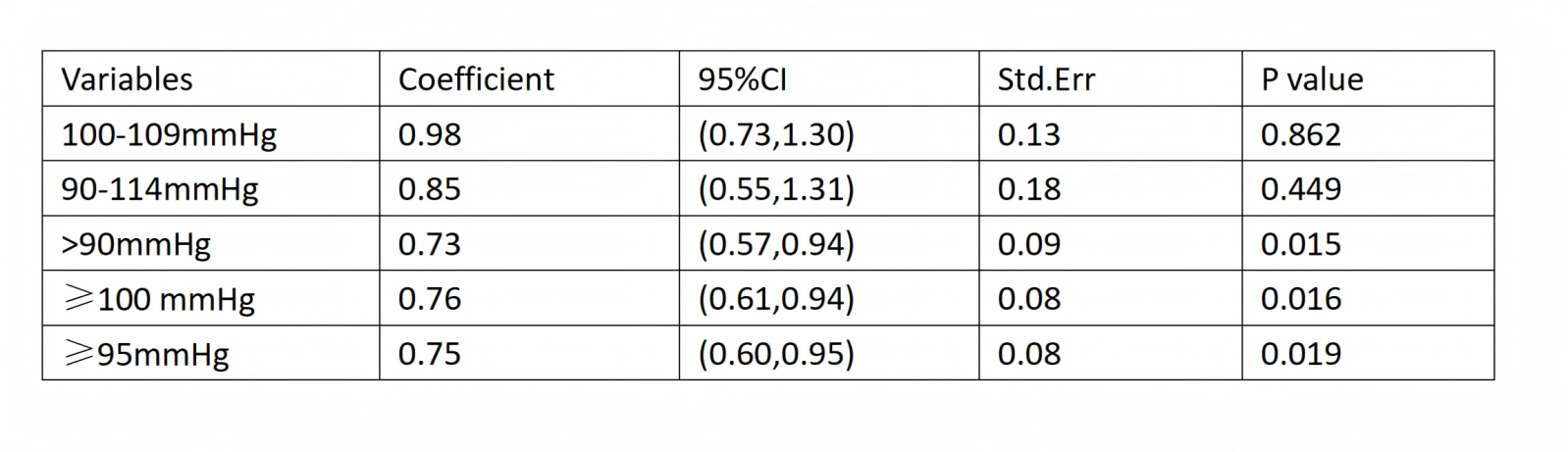


Table17:Blood pressure control rates Regression - Baseline(DBP)


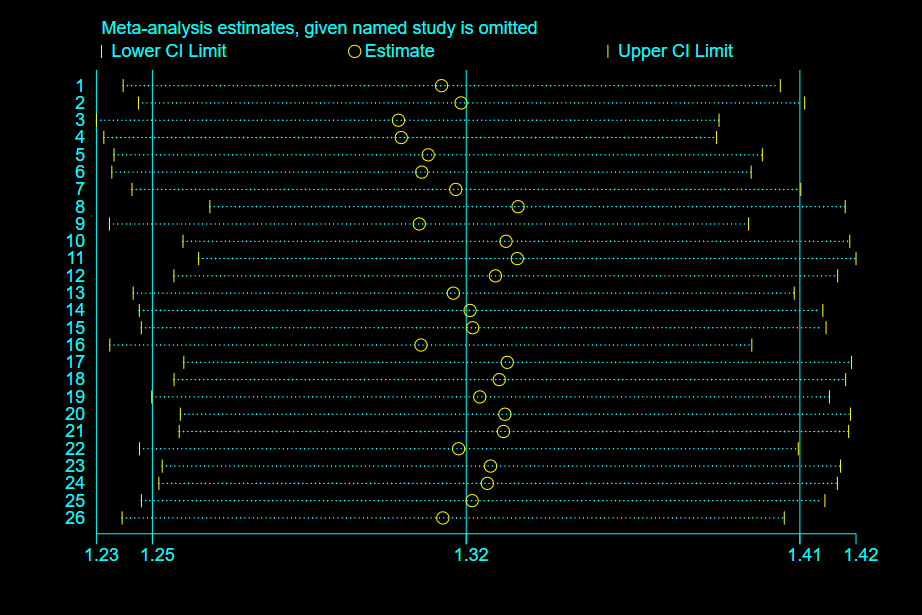


Figure 15:Blood pressure control rates(excluding the 1988 study)-sensitivity analysis


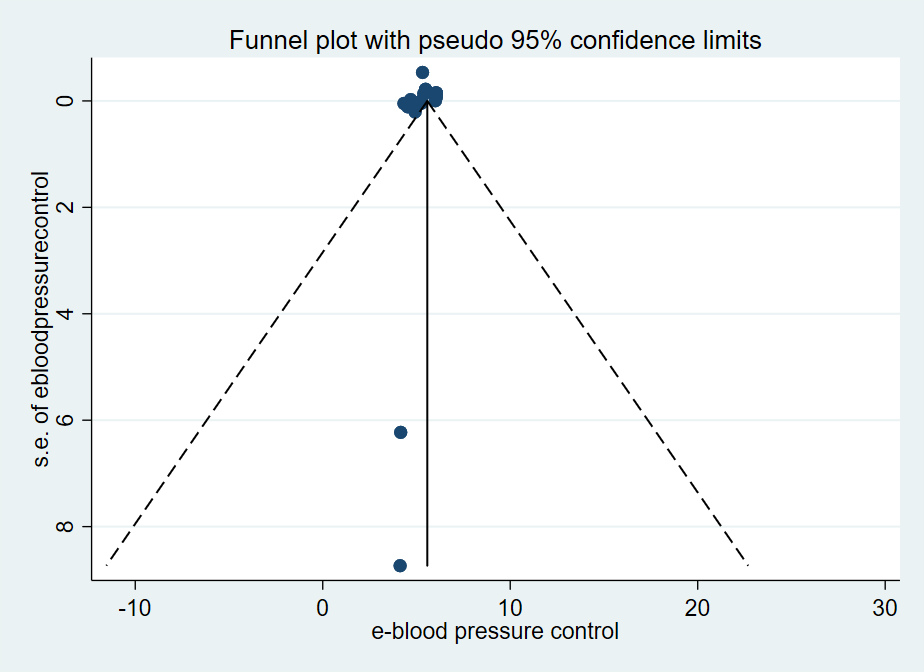


Figure 16:Blood pressure control rates(excluding the 1988 study)-Funnel Graph


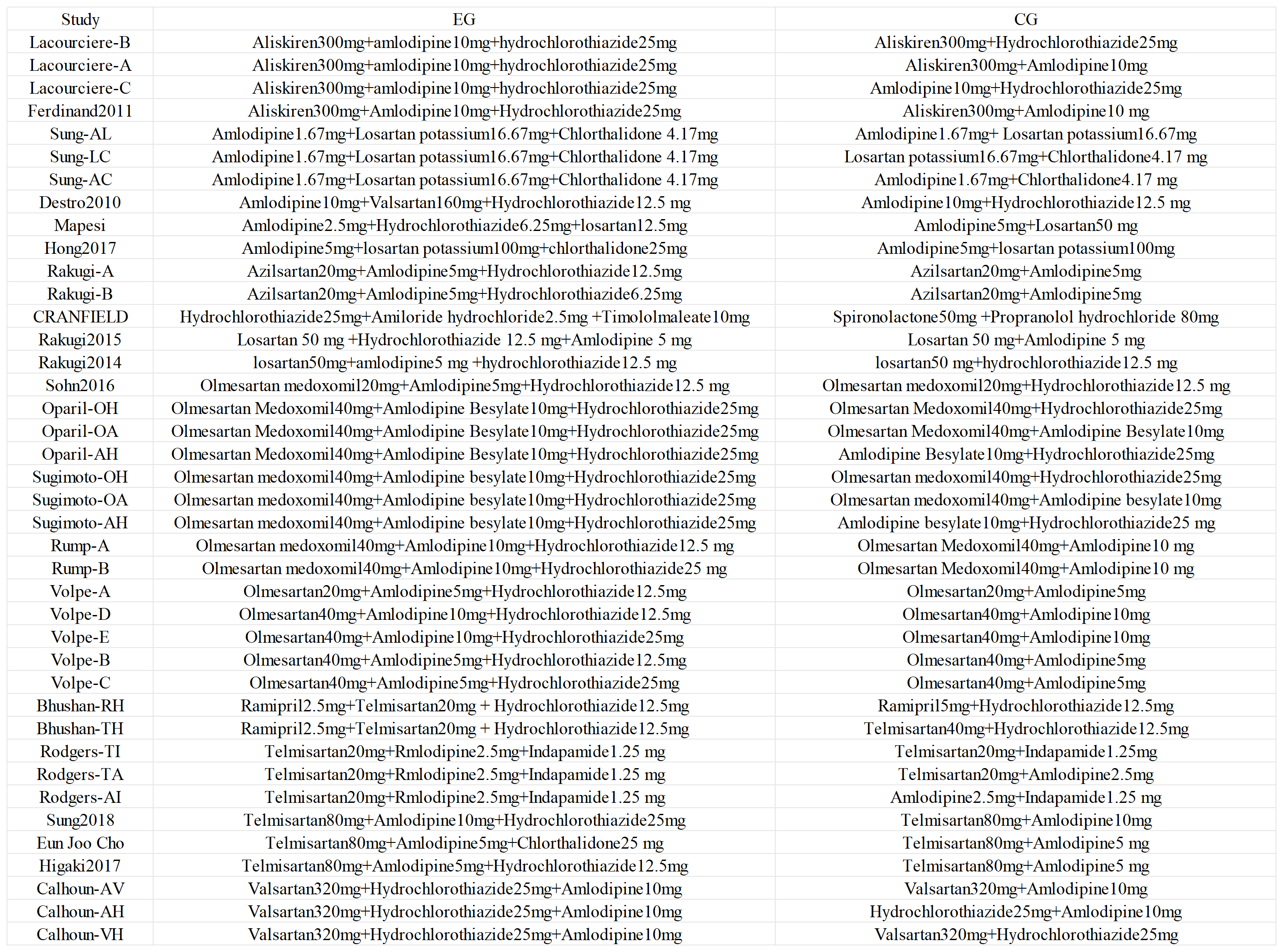


Research ID Number
